# Zinc Gallium Oxide—A Review from Synthesis to Applications

**DOI:** 10.3390/nano10112208

**Published:** 2020-11-05

**Authors:** Mu-I Chen, Anoop Kumar Singh, Jung-Lung Chiang, Ray-Hua Horng, Dong-Sing Wuu

**Affiliations:** 1 Department of Materials Science and Engineering, National Chung Hsing University, Taichung 40227, Taiwan; chenmy1989@gmail.com (M.-I.C.); anoop.scns@gmail.com (A.K.S.); cjunglung@gmail.com (J.-L.C.); 2 Department of Electronics Engineering, National Chiao Tung University, Hsinchu 30100, Taiwan; 3 Innovation and Development Center of Sustainable Agriculture, National Chung Hsing University, Taichung 40227, Taiwan

**Keywords:** ZnGa_2_O_4_, spinel structure, wide-bandgap, photodetector, phosphor, gas sensor

## Abstract

Spinel ZnGa_2_O_4_ has received significant attention from researchers due to its wide bandgap and high chemical and thermal stability; hence, paving the way for it to have potential in various applications. This review focuses on its physical, optical, mechanical and electrical properties, contributing to the better understanding of this material. The recent trends for growth techniques and processing in the research and development of ZnGa_2_O_4_ from bulk crystal growth to thin films are discussed in detail for device performance. This material has excellent properties and is investigated widely in deep-ultraviolet photodetectors, gas sensors and phosphors. In this article, effects of substrate temperature, annealing temperature, oxygen partial pressure and zinc/gallium ratio are discussed for device processing and fabrication. In addition, research progress and future outlooks are also identified.

## 1. Introduction

In recent years, zinc gallium oxide (ZnGa_2_O_4_) with a spinel structure has attracted much attention, because it has a wide-bandgap, high transmittance against the ultraviolet (UV) region, and stability under vacuum [1]. Furthermore, it is a promising phosphor, because of its chemical and thermal stability and its blue emission when irradiated by UV light or subjected to low-voltage electrons [2]. ZnGa_2_O_4_ was first discovered as a new UV-transparent electronic conductor by Omata et al. in 1994 [3]. It belongs to the family of transparent semiconducting oxides and has a bandgap of 4.4–5.0 eV [4,5]. Bulk ZnGa_2_O_4_ single crystals possess high electron mobility, ∼100 cm^2^ V^−1^ s^−1^ at a high free electron concentration (mid 10^19^ cm^−3^), which confirms that the network of edge-shared GaO_6_ octahedra possesses a wide bandgap and high mobility, as in the case of *β*-Ga_2_O_3_ [6]. ZnGa_2_O_4_ is a promising semiconductor oxide because of its wide-bandgap, which makes it an ideal candidate for the fabrication of solar-blind photodetectors (PDs) due to its lack of sensitivity to light with a wavelength above 280 nm. These photodetectors are of significant use in the detection of trace dangerous pollutants in a confined space, such as nitric oxide, due to strong absorption in the 200–250 nm region. This wide-bandgap material is attracting significant attention due to the possibility of its use not only in high-powered devices, but also in UV-transparent devices—especially for applications in conjunction with biological samples—because absorption peaks in deoxyribonucleic acid (DNA) lie in the UV spectrum [6]. Recently, Chikoidze et al. demonstrated the p-type ZnGa_2_O_4_ semiconductor (∼5 eV), which can pave the way for bipolar oxide energy electronics (it can save the loss from switching and conversion of electrical energy), as they would join the required qualities of sustaining large electrical fields in p-n junctions in the off-state together with low losses in the on-state [7]. Besides this, wide-bandgap semiconductors enhance the efficiency of power-conversion stages and these are an alternative for silicon in the manufacturing of voltage converters, power MOSFETs, and high-efficiency Schottky diodes, which can be further used in electric vehicles and hybrid electric vehicles. This article provides detailed information regarding ZnGa_2_O_4_ bulk as well as thin films, which will be significant to the research community in the future. The history of ZnGa_2_O_4_ dates back to 1970, when van den Boom et al. [8] published an article about the electron spin resonance (ESR) spectrum with Ga substituted by Cr^3+^ ions in the spinel ZnGa_2_O_4_ described by a uniaxial spin-Hamiltonian. Early publications focused on the ESR of chromium doped ZnGa_2_O_4_ in the 1970s, as in References [9,10,11].

**Figure 1:**
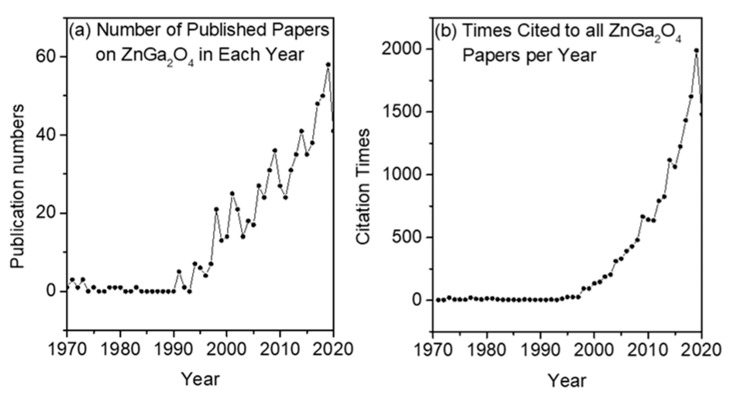
Statistic line graph about ZnGa_2_O_4_ publications (**a**) Number of publications from 1970 to 2020/10/22. The total number of the publications is 732. (**b**) Citation times to all ZnGa_2_O_4_ papers from 1971 to 2020/10/22. The total number of citations is 16,567.

Later research about ZnGa_2_O_4_ was gradually developed in the 1990s, as shown in Figure 1a [12]. During this decade, oxide phosphors that emit multiple colors had been investigated widely for their applications in field emission display (FED), vacuum fluorescent display (VFD) and plasma display panels, and thin-film electroluminescent devices. Oxide phosphors exhibit higher chemical stabilities and thermal stabilities in a high vacuum than sulfide phosphors [13], and various luminescent properties such as photoluminescence (PL), cathodoluminescence (CL), and electroluminescence (EL) were investigated. Among them, PL layers and EL systems prepared by different methods have been systematically studied for the fabrication of flat panel displays, monitoring screens, and lighting systems [14]. According to the electrically conductive properties of ZnGa_2_O_4_, it can also be used as a blue-emitting phosphor for VFDs and FEDs utilizing low-voltage cathodoluminescence [15]. In addition, the mechanoluminescence properties of ZnGa_2_O_4_: Mn were also investigated [16].

ZnGa_2_O_4_ is an attractive material for the phosphor of fluorescent lamps [17]. ZnGa_2_O_4_ phosphors possess intrinsic blue emission characteristics, and the doping of metal ions may lead to a shift in its emission wavelength [18] and manifest various functional properties that can be applied to optoelectronic devices [19]. Recently, ZnGa_2_O_4_ thin films have been investigated for deep-ultraviolet photodetectors [20]. Porous ZnGa_2_O_4_ microspheres are synthesized by a facial glucose-mediated microwave hydrothermal method followed by annealing, in order to investigate the photocatalytic degradation of gas-phase aromatic pollutants, where porous ZnGa_2_O_4_ microspheres confirmed a much higher activity and stability than TiO_2_ under 254 nm UV irradiation [21]. Another possible application of the ZnGa_2_O_4_ materials, excluding low-frequency vibrational harvesting, could be a pressure sensor or strain gauge, due to the good linearity of the electrical parameter dependences on the strain [22].

Rare-earth ions are widely used as activators that can be doped in ZnGa_2_O_4_ due to their high fluorescence efficiencies and very narrow line fluorescence bands [23]. Thus, the ZnGa_2_O_4_: Yb^3+^, Er^3+^, Tm^3+^ upconversion phosphors with the doping of Sn^4+^ and Ge^4+^ ions were proposed for color modification applications [24]. In addition to rare-earth ions, transition metal ions are often doped in ZnGa_2_O_4_. In the early 1990s, PL properties of cobalt doped ZnGa_2_O_4_ were observed and analyzed by Abritta and Blak in 1991 [25]. Co^3+^ doped ZnGa_2_O_4_ phosphor shifted its emission wavelength to 660 nm and emitted reddish-orange light [26]. After that, PL of manganese activated ZnGa_2_O_4_ was investigated by Shea in 1994 [27]. The PL, CL, and EL of the ZnGa_2_O_4_: Mn phosphor has been frequently studied for its application in emissive devices onwards [15]. Note that manganese doped zinc gallate (ZnGa_2_O_4_) is a well-known phosphor which shifts the emission wavelength of zinc gallate phosphors from the blue intrinsic region to the green region (510 nm) [28].

Cr-doped ZnGa_2_O_4_ is a red phosphorescence material that has potential in vivo imaging [28,29]. Germanium or tin substitution into the Cr-doped ZnGa_2_O_4_ results in the improvement of the red long-lasting phosphorescence [30] and ZnGa_2_O_4_: Mn^2+^ also possesses long-lasting characteristics [31]. The grain size and crystal structure have been considered as extraordinary parameters in studying the luminescence characteristics of phosphors [32].

The low voltage cathodoluminescence (LVCL) of ZnGa_2_O_4_ phosphor was first investigated by Itoh in 1991, due to its application to VFDs [33,34]. Further investigations about LVCL were also published by Hsieh et al. [33] in 1994, where they used radio frequency magnetron sputtering to prepare ZnGa_2_O_4_ thin films. Furthermore, the thin film of ZnGa_2_O_4_ could be used as a phosphor in FED because of its good cathodoluminescence characteristics at low voltage [35]. Since light emission of FED depends on LVCL characteristics, hence FED was found as an emerging candidate for replacing typical cathode ray tubes, which were earlier used in flat panel displays [35]. Manganese doped zinc gallate was also found to be an LVCL phosphor which emits green color (510 nm) [36].

ZnGa_2_O_4_ is a wide-bandgap semiconductor and its material properties are compared with other wide-bandgap semiconductor material properties, which are shown in Table 1. After 2000 A.D., the number of publications on ZnGa_2_O_4_ was significantly increased onwards and the total number of citations toward ZnGa_2_O_4_ was also increased as shown in Figure 1b [12]. It is not possible to include every aspect of ZnGa_2_O_4_ for this review paper. Hence, only some important aspects are discussed here. Section 1 is an introduction which deals with the historical evolution of ZnGa_2_O_4_. Section 2 represents the basic properties of ZnGa_2_O_4_ where the crystalline structure of ZnGa_2_O_4_ will be elucidated and the band structure will be calculated by the first principle method. Section 3 contains the growth mechanisms of bulk ZnGa_2_O_4_. Section 4 discusses the growth mechanisms of zinc gallate thin films where the effects of various parameters such as substrate, operating temperature, oxygen partial pressure, and annealing temperature will be investigated separately. Section 5 contains the applications of ZnGa_2_O_4_ where special focus is given to deep-ultraviolet photodetectors. Considering all the above sections, the final section deals with concluding remarks and perspectives.

## 2. Basic Properties of ZnGa_2_O_4_

Before introducing the different phases of ZnGa_2_O_4_ and its growth mechanism and applications, some fundamental properties of ZnGa_2_O_4_ are important to discuss, because these are the key to understanding ZnGa_2_O_4_. Several basic properties of ZnGa_2_O_4_ contain its crystalline structure, mechanical properties, band structure, and bond distance along with electronic properties, which will be discussed as follows.

### 2.1. Crystalline Structure of ZnGa_2_O_4_

The spinel structure of AB_2_O_4_ crystals was first determined in 1915 by Bragg and Nishikawa [41]. Various spinel oxides have significant optoelectronic properties, because they are wide-bandgap materials [42], and ZnGa_2_O_4_ is one of them. ‘Spinel’ gets their name from a red gemstone which can occur in different shades of blue, purple, and pink [43].

**Figure 2:**
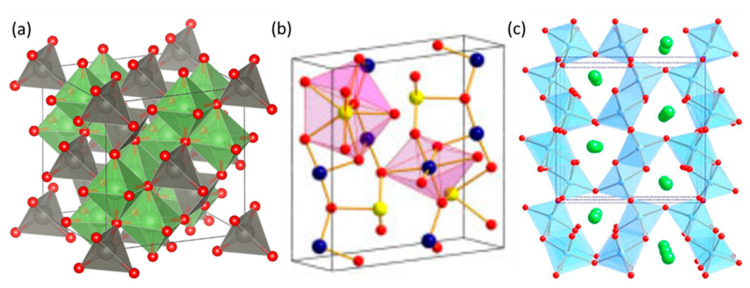
(**a**) Cubic spinel structure of ZnGa_2_O_4_, displaying tetrahedrally coordinated Zn atoms (in gray) and octahedrally coordinated Ga atoms (in green). Oxygen atoms are shown in red [44] (with copyright permission from American Chemical Society, 2020). (**b**) Schematic view of the orthorhombic hexagonal phase (CaMn_2_O_4_-like) of ZnGa_2_O_4_. The atom color palettes for Zn, Ga, and O are yellow, blue, and red, respectively [46] (with copyright permission from John Wiley and Sons, 2020). (**c**) The structure of CaTi_2_O_4_ showing TiO_6_ octahedra in blue and calcium ions as green spheres [47] (with copyright permission from Elsevier, 2020).

The spinel structure material ZnGa_2_O_4_ consists of ZnO and Ga_2_O_3_, hence termed as binary compound oxide [35]. The spinel crystal is cubic symmetric, with a space group of Fd$\bar{3}$m, with oxygen atoms in cubic close packing and cation atoms in face-centered cubic packing. The spinel structure is of two types: normal spinel and inverse spinel, and ZnGa_2_O_4_ can possess both types of these structures. In the normal spinel, divalent Zn cations are tetrahedrally coordinated with oxygen atoms while the Ga cations occupy octahedral sites [44], as shown in Figure 2a. The divalent Zn cations fill one eighth of the tetrahedral sites and the trivalent Ga cations fill one half of the octahedral sites in the normal spinel crystal.

Another spinel structure is known as the inverse spinel structure, in which Zn cations and half of the Ga cations occupy the octahedral sites and the other half of the Ga cations occupy the tetrahedral sites. Intermediate atomic distributions can also occur often and these are characterized by the degree of inversion [45]. The normal spinel structure is preferred to the inverse spinel structure. Hence, the special focus is given to normal spinel structure here [4].

High-pressure XRD studies reveal that ZnGa_2_O_4_ undergoes two transitions. The first transition occurs in ZnGa_2_O_4_ from the cubic spinel (Fd$\bar{3}$m) toward the orthorhombic CaMn_2_O_4_-type structure (*Pbcm*) under hydrostatic pressure of 33.4 GPa, which was studied by Moreno et al. [46] where they concluded that phonon frequencies from the orthorhombic CaMn_2_O_4_-type structure (*Pbcm*) phase of ZnGa_2_O_4_ have a noteworthy change at 42.5 GPa. CaMn_2_O_4_ (*Pbcm*) and CaTi_2_O_4_-type (*Cmcm*) structures exist simultaneously at this pressure, but the CaTi_2_O_4_-type (*Cmcm*) structure is more stable than the CaMn_2_O_4_ (*Pbcm*) one. Therefore, a second order phase transition occurs at this pressure of 42.5 GPa. The structures of the aforementioned phases above are shown in Figure 2a–c. In order to focus more attention on the understanding of the high-pressure properties of ZnGa_2_O_4_ cubic spinels, the mechanical properties of ZnGa_2_O_4_ under pressure will be discussed in the next section.

### 2.2. Mechanical Properties of ZnGa_2_O_4_ under External Pressure

Researchers have been significantly interested in the lattice dynamics of spinels, experimentally as well as theoretically [48]. AB_2_O_4_ compounds are investigated widely for the aforementioned purpose, because they have structural dependence under pressure. This material can be found in many geological settings of the earth’s crust and mantle, in lunar rocks and meteorites [49]. The zinc atoms are located at the Wyckoff positions, 8a (1/8, 1/8, 1/8) tetrahedral sites, while the gallium atoms take position at the 16d (1/2, 1/2, 1/2) octahedral sites and the oxygen atoms are at 32e (*u*, *u*, *u*), where *u* is approximately 1/4 [45]. In crystallography, a Wyckoff position is a point belonging to a set of points for which site symmetry groups are conjugate subgroups of the space group. Lattice parameter ‘*a*’ and the internal parameter ‘*u*’ characterize the spinel crystal structure.

Theoretical calculations based on a generalized gradient approximation (GGA) exchange-function over-estimates the lattice parameters and under-estimates the bulk modulus of experimental data for spinels, while local-density approximations (LDA) exhibit the opposite behavior [45]. In addition to the LDA method, Zhang et al. took the external factor and purity of the materials into account. Therefore, their calculated results (*a* = 8.275 Å, *u* = 0.261) were found to match with the experimental models (*a* = 8.334 Å, *u* = 0.262) [45]. The lattice constants of ZnCr_2_O_4_ and ZnGa_2_O_4_ are almost the same but larger than that of ZnAl_2_O_4_. It seems that the variation of the lattice constants with the different electronic shells is larger than that with the same electron shells. Additionally, *u* decrease with the increase in the size of the trivalent cation [45]. Dixit et al. used plain LDA in all cases where all the calculated results were found to be in agreement with the experimental models [4]. Bouhemadou et al. reported that the lattice constant has a negative slope relationship with pressure [50]. Furthermore, in their work, the internal parameter ‘*u*’ approached 0.25 under the effect of pressure, which revealed that the spinels expand their effort to get the ideal structure.

ZnGa_2_O_4_ is compressible because the lattice constant and the internal parameter are functions of pressure. Elastic properties such as elastic constants (C_ij_) and bulk modulus (B_0_) are worthy to discuss here. When these parameters are small, the bulk solid is easy to compress. The elastic properties of a solid play an important role in determining the interatomic bonding, phonon spectra, and equations of state [50]. The elastic constant is the ratio between applied stress and computed strain. Stress and strain possess six components, respectively, in which each of them has three tensile and three shear components. The relation between the stress and the strain can be expressed as σ_i_ = C_ij_ε_j_, where i, j = 1~6, σ is stress, and ε is strain. If there is any symmetry, some of these components may be equal or zero. Hence, a cubic crystal possesses only three different symmetry elements (C_11_, C_12_ and C_44_), each of these represents three equal elastic constants (C_11_ = C_22_ = C_33_; C_12_ = C_23_ = C_31_; C_44_ = C_55_ = C_66_) while others can be fixed a zero.

Bouhemadou et al. concluded that the bulk modulus (B_0_) and elastic constants (C_ij_) are linear positive correlations to the pressure for ZnGa_2_O_4_ [50]. Their results may be considered as reliable predictions of the pressure dependence of the elastic properties [50] for the ZnGa_2_O_4_ material, excluding low-frequency vibrational harvesting, which could be a pressure sensor or strain gauge, due to the good linearity of the electrical parameter dependences on the strain [22]. The values for the elastic constants were C_11_ = 228 GPa, C_12_ = 120 GPa, C_44_ = 107 GPa, and bulk modulus B_0_ =156 GPa when the pressure was subjected to zero. From the elastic constants and bulk modulus aforementioned above, the anisotropy parameter was found to be 1.98 using A = 2C_44_/(C_11_ − C_12_) where ‘A’ is known as the anisotropy parameter, which indicates that ZnGa_2_O_4_ was highly anisotropic [50].

### 2.3. Band Structure of ZnGa_2_O_4_

ZnGa_2_O_4_ is a wide-bandgap semiconductor [45], and is found to be considerable in many optical applications. One of the important applications of ZnGa_2_O_4_ is as a deep-ultraviolet photodetector, which will be mentioned in the fifth section. Luminescence, in a particular spectral region, can be obtained by using dopants in the ZnGa_2_O_4_ compound [51]. The bandgap can be obtained by using reflectance spectroscopy [52]. However, the bandgap obtained by a variety of first principle numerical calculations is compared with experimental values.

**Figure 3:**
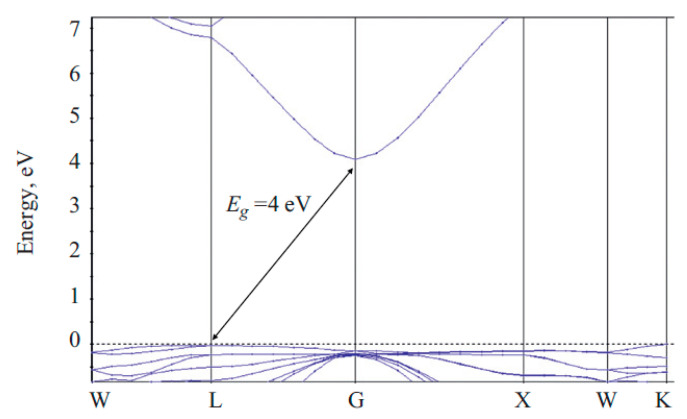
Calculated band structure of ZnGa_2_O_4_ [51] (with copyright permission from Elsevier, 2020).

ZnGa_2_O_4_ has a bandgap calculated to be 2.79 eV (direct) [53], 2.73 eV (indirect) [45] 2.571 eV (indirect) [51] and 2.82 eV (indirect) [4] by using the density functional theory (DFT) method. However, this result is quite different to the experimental value 4.0 eV [51] or 4.4–5.0 eV [4]. Such an underrated calculated bandgap is related to well-known DFT limitations [51]. In order to overcome such a discrepancy, Brik [51] used a scissor operator which added 1.5 eV to the originally calculated bandgap. Then, the indirect bandgap increased from 2.571 eV to 4.071 eV, approaching the experimental value. The corrected band structure is shown in Figure 3.

Furthermore, Dixit et al. [4] revealed that the DFT–LDA bandgap of ZnGa_2_O_4_ was underrated, because a significant p–d hybridization took place between the Zn-d and O-p orbitals. They used the GW and modified Becke–Johnson (MBJ) methods to correct the bandgap at the Γ point with the Zn^20+^ and Ga^21+^ pseudopotentials, obtaining the bandgap of 4.57 and 4.71 eV, respectively. These two results are close to the experimental values of bandgap 4.4–5.0 eV.

### 2.4. Bond Distance and Its Electronic Properties

The experimental bond distance of Zn-O is 1.98 Å and that of Ga-O is 1.99 Å, while the calculated bond distance of Zn-O is 1.95 Å and that of Ga-O is 1.98 Å [45]. These results are slightly underrated. Zhang et al. reported that the bond distance of Zn-O decreases faster than that of Ga-O under pressure, which means that Zn-O bond is softer than the Ga-O bond. The bond distances d_Zn−O_ and d_Ga−O_ get far away from each other with the increase in pressure [45]. Different studies have found that the calculated bond distances of Zn-O and Ga-O were 1.99713 Å and 2.01716 Å, respectively [51].

When bonded together, the Mulliken charges of individual atoms represent the re-distribution of the electron state densities between the ions. Mulliken charges versus pressure are noteworthy to study. Increased positive value of the Mulliken charge for Zn ions in ZnGa_2_O_4_ can be attributed to the strong mixture with surrounding oxygen ions. Brik reported that the absolute values of the Mulliken charges of oxygen and gallium are increasing with pressure, while the absolute values of the Mulliken charges of zinc decrease with pressure [51]. This phenomenon is attributed to a shift of the negative-charged electron density from gallium ions toward oxygen ions and from oxygen ions toward zinc ions with the former shift dominating over the later one. Mulliken bond order provides additional information about the nature of the chemical bond between the atoms. Higher values of the bond orders represent the covalent character of the bond, while lower values of the bond orders represent the ionic nature of the bond. Ga–O is more ionic than Zn–O, or Zn–O is more covalent than Ga–O. An increase in bond orders or increase in covalency with the increase in pressure can be observed, and it has no relation with the nature of the bonds [51].

Electron distributions around atoms of ZnGa_2_O_4_ are not spherical symmetric. Electron localization function contour plots give a clear interpretation of bonding characteristics of the system. Sampath et al. found that the electron localization function contours of ZnGa_2_O_4_ represent a strong localized region around the oxygen and the significant distortions due to the participation of the Zn 3d orbitals and the localized Ga 3d orbitals could be observed in the ZnGa_2_O_4_ bonding [53]. This result illustrates the covalency of ZnGa_2_O_4_.

## 3. Bulk Growth Mechanisms

Research on binary compounds focuses on the availability of bulk crystals—that are thermally stable at high temperatures and which possess high melting points. ZnGa_2_O_4_ is a potential candidate in the aforementioned perspective. There are various methods to synthesize the bulk ZnGa_2_O_4_ crystals, such as the conventional solid-state method, flux crystal growth method, Czochralski method, laser heated pedestal growth method, and hydrothermal method, etc. These bulk crystals are favorable because these can be utilized as a substrate for the epitaxial growth and related devices.

### 3.1. Solid-State Method

The solid-state method is used for synthesizing micro/nanostructures. Reaction among solids does not take place at room temperature. Hence, high temperature (1000–1500 °C) is required to initiate the chemical reaction between them. This method is typically a two-step process which is promising for the easy synthesis of compounds. Mixing/milling of starting materials takes place with the help of a mixing agent followed by the thermal treatment above 1000 °C. Higher temperature leads to the volatilization of the material in the compound process. This is a low-cost method apart from its necessity of higher temperature for sintering.

Can et al. [54] prepared the ZnGa_2_O_4_ particles by using a traditional solid-state method with zinc oxide, zinc hydroxide, gallium oxide hydroxide, and H_2_O as the starting precursors, and the sample was milled under the thermal treatment at 1200 °C for several hours, which leads to the volatilization of zinc oxide and hence gives non-stoichiometric composition [55]. Thermal treatment is required at a higher temperature to achieve the homogeneity in the sample.

### 3.2. Flux Growth Method

The flux method is a technique for crystal growth where the resulting substances are dissolved in a solvent (flux). This flux also requires a high temperature (1000–1500 °C). Crystal formation in this technique can take place either by spontaneous nucleation or may be initiated by employing a seed. This method is favorable when crystals are required to be free from thermal strain. The advantage is that the natural facets of the grown crystals occur, which helps to measure such crystals precisely.

Most flux methods synthesize small crystals, such as those of Chase and Osmer [56], who obtained ZnGa_2_O_4_ crystals in the size range of 3–6 mm using PbO-PbF_2_-B_2_O_3_ flux. Vanderstraten et al. [57] synthesized large ZnGa_2_O_4_ crystals—up to 10 mm along the edge—using the same flux with the addition of SiO_2_ at the cooling rate of 0.5 °C/h. However, the PbF_2_ rich flux method led to the poor quality of single crystals, because of the contamination from Pb in the ZnGa_2_O_4_ crystal lattice. Yan et al. [58] synthesized similar ZnGa_2_O_4_ crystals—having a 10 mm edge length—by slowly cooling a PbF-free and PbO-B_2_O_3_ flux. As grown ZnGa_2_O_4_ samples are electrical insulators; hence, annealing with hydrogen makes the bulk ZnGa_2_O_4_ conductive [59].

### 3.3. Czochralski Method

The Czochralski method is widely used in manufacturing semiconductor devices, to grow a single crystal silicon ingot. Circular wafers are obtained by cutting the pieces of the silicon crystals into thin films, and then the circular wafers are implanted into small silicon chips from which large-scale integrated silicon chips are made. The parameters associated with this method are the maximum crystal pulling rate and crucible rotation rate, which are 10 mm/min and 35 rpm, respectively. This method requires the use of crucibles, which can lead to contamination from the melting of the crucible at the high temperature. Generally, ingots can obtain lengths and diameters in the range of 1 to 2 m and 4 to 6 inches, respectively. However, the magnetic field enables the ingot’s diameter up to 12 inches nowadays.

Zinc oxide and gallium oxide are thermally unstable at high temperature, which leads to highly anomalous decomposition and evaporation in the formation of ZnGa_2_O_4_ crystals. Hence ZnGa_2_O_4_ crystal requires the precise stoichiometric composition. To overcome this problem, Galazka et al. [38] synthesized the ZnGa_2_O_4_ crystals from the melt using the Czochralski and the vertical gradient freeze method simultaneously, which helped them in obtaining defined shape, size, and reduced dislocation densities in the crystals. The molten single-crystalline spinel ZnGa_2_O_4_ crystal was thermally stable up to 1100 °C. Such a grown-up mechanism suggested that ZnGa_2_O_4_ can be employed as a lattice-matched substrate for epitaxial magnetic ferrite spinel films. A high melting point of 1900 ± 20 °C was found for ZnGa_2_O_4_. To avoid the decomposition of oxide, control of the melt thermodynamics was necessary. As a result, ZnGa_2_O_4_ was found to be a cubic system and had no specific crack planes, which enabled the development of a large volume of crystals and easy wafer fabrication.

### 3.4. Laser Heat Pedestal Growth Method

The laser heat pedestal growth (LHPG) method allows the formation of crystals with micron-size diameters with the help of CO_2_ or YAG lasers. The laser is moved along the crystal, so the molten area melts the impure solid on its front edge and leaves a wake of pure solidified material behind it. LHPG is a crucible-free method so there is no existence of contamination [60]. This method is favorable in the growth of single-crystal fibers. This method synthesizes single crystals that have low stress and high purity, which leads to the possibility of synthesizing a large volume of crystals with higher melting points. It is applicable no matter whether the melt evaporates congruently or incongruently. However, crystals are difficult to synthesize in the case of incongruent melting because of the large surface area to volume ratio of the melt. The growth rate is slow due to the steep axial temperature gradients (1000 °C/cm) [61,62,63].

Single crystal fibers of ZnGa_2_O_4_ were prepared by using the LHPG method in [64]. A 0.5% concentration of Mn^2+^ in ZnGa_2_O_4_ was introduced to study the cathodoluminescence properties. The highly volatile nature of zinc oxide and gallium oxide at 1100 °C causes major problems in fiber growth, and the higher surface to volume ratio of the molten zone of fiber and the vaporization of constituents in the melt can be expected to change the desired phase condition and inhibit the growth of the crystal. Therefore, a lower laser power and faster-pulling speed were used by them during crystal growth to reduce the effect of vaporization.

### 3.5. Hydrothermal Method

Melt grown methods require the necessity of high temperature, which in turn leads to the non-stoichiometric composition and the impurity phases. However, the hydrothermal method can synthesize single crystals at very low temperatures, and can be used widely for the synthesis of oxide materials. It has the following advantages:

1. It can synthesize oxide materials in crystalline phases that are not found to be stable at the melting point.
2. Crystal growth occurs with lower thermal strain; hence it has lower dislocation density than the melt grown methods where the large value of temperature gradient is required.
3. A large volume of high-quality crystal can be obtained by keeping control of composition.

**Figure 4:**
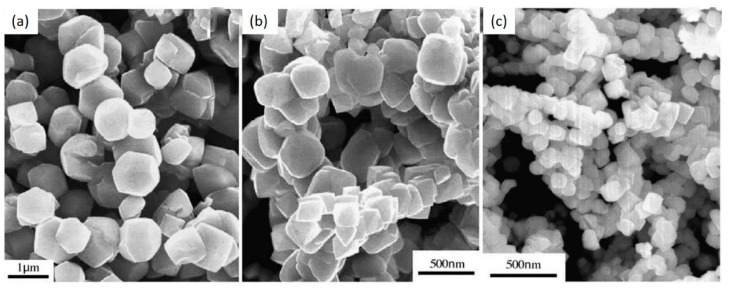
SEM image of ZnGa_2_O_4_ crystals with different pH values (**a**) pH = 12, (**b**) pH = 10, (**c**) pH = 8 [67] (with copyright permission from Elsevier, 2020).

The hydrothermal method requires an autoclave and a steel pressure vessel in which a nutrient is supplied along with water in the temperature range between 100 and 374 °C, with elevated pressure up to 150 MPa [65]. The facing walls of the growth chamber require an autoclave temperature gradient which maintains the balance between them. This temperature gradient further helps to dissolve the solute nutrient at the hot end, and it is deposited on the seed crystal at the cooler end, which leads to the desired morphology. Further, controlled particle morphology, phase homogeneity, and decrease in agglomeration between particles have been accepted as the usefulness of this method [66]. ZnGa_2_O_4_ crystals were reported using this method by Chen et al. [67] where they have examined the structural properties with the different pH values. ZnGa_2_O_4_ spinel crystals with various morphology were prepared using zinc salt, gallium oxide hydroxide, gallium sulfate, and gallium oxide. The mixture of the above reagents was prepared under the various pH value in the range of 8–12. This process further required the solution to keep in an autoclave for 8–24 h at 180 °C. As grown particles in Figure 4a–c exhibited the cubic shape and the non-uniform polyhedron shape when the pH value was increased from 8 to 12, respectively [67].

**Figure 5:**
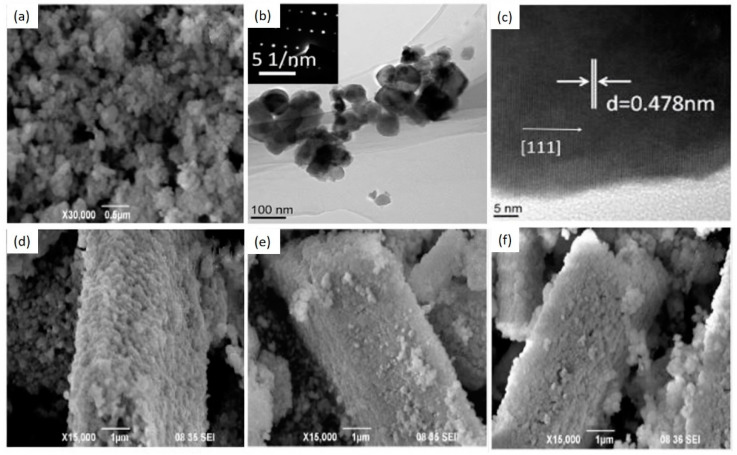
SEM and TEM images of ZnGa_2_O_4_ crystallites were prepared at different thermal hydrothermal temperatures. (**a**) SEM image of ZnGa_2_O_4_ crystallites prepared at 160 °C, (**b**) TEM image of ZnGa_2_O_4_ powders obtained at 160 °C. The inset shows the SAED pattern from individual ZnGa_2_O_4_ spherical particle. (**c**) The HRTEM picture of ZnGa_2_O_4_ crystallites obtained at 160 °C, (**d**–**f**) SEM images of ZnGa_2_O_4_ crystallites obtained at 170, 180 and 200 °C [68] (with copyright permission from Elsevier, 2020).

Liu et al. [68] studied the influence of temperature on the morphology of ZnGa_2_O_4_ crystallites synthesized by the hydrothermal method. ZnSO_4_.10H_2_O and Ga_2_O_3_ were taken in certain molar ratios and mixed with deionized water. The pH value of the solution was maintained in the range of 8–14 using NaOH. The solution was contained in the vessel and heated at 160–200 °C for 4 h with constant rotation and then cooled down to reach the room temperature. The products were washed using deionized water and ethanol, then dried at 80 °C for 3 h. Figure 5 shows the SEM and TEM images of ZnGa_2_O_4_ crystallites prepared at different hydrothermal temperatures. Agglomerated nano-spherical particles were observed at the hydrothermal temperature of 160 °C, as shown by the SEM image in Figure 5a. These nano-spherical particles were found to be single-crystalline as shown by the TEM images in Figure 5b,c where the d-spacing between the adjacent planes for (111) lattice plane was found to be 0.478 nm. Figure 5d–f shows the SEM images at different hydrothermal temperatures of 170, 180 and 200 °C. With the increase in temperature, ZnGa_2_O_4_ spherical particles converted into a large size aggregated cuboid-shaped morphology and denser surface, due to the recrystallization of ZnGa_2_O_4_ nano-grains.

The following Table 2 summarizes all the methods which are discussed above for the bulk growth of ZnGa_2_O_4_ along with their characteristics:

## 4. Thin Films of ZnGa_2_O_4_

The thin-film properties of a material are quite different than the properties of bulk powder; hence, thin films have significant characteristics such as better thermal stability, reduced outgassing [71,72], and a better lifetime for devices [28]. Phosphors based on thin film possess lower luminescent intensity than the bulk ones. The reason behind their lower luminescent intensity is the optical confinement of light due to planar interfaces [73]. Enhancing crystallinity of thin films then becomes an important issue and as a result of this issue, investigation of various parameters such as growth temperature, substrate species, oxygen pressure, and annealing temperature has taken place [74].

ZnGa_2_O_4_ thin films are synthesized by various techniques such as radio frequency (RF) magnetron sputtering, chemical vapor deposition (CVD), sol-gel processing, and pulsed laser deposition (PLD) [75]. Among them, PLD and sputtering belong to physical vapor deposition, while CVD consists of metal-organic chemical vapor deposition (MOCVD) and mist CVD. MOCVD has been found to be favorable due to its high deposition rate and large-area uniformity, which leads to single-crystalline structures [76].

PLD exhibits significant advantages, such as the stoichiometry of deposited films close to that of the target, high deposition rate, low contamination level [71], precise arrival rates of atoms for compound films, and the ability to operate in high-pressure reactive gases [77]. In fact, RF magnetron sputtering has been the most successful among the various growth techniques for wide-area applications due to its easy controllability of the growth parameters, excellent packing density [74], strong adhesion, excellent film thickness uniformity, and relatively low running cost [78]. Polycrystalline or amorphous film structures are usually obtained by the RF magnetron sputtering method, which significantly affects the optoelectronic properties of the material [74].

Crystalline orientation is often identified by X-ray diffraction (XRD) pattern, while crystallinity, often related to stoichiometric properties of ZnGa_2_O_4_, the ratio of Zn/Ga, is proportional to the luminescent intensity. Better luminescent characteristics can be obtained by giving significant attention to growth conditions [32]. Thus, analyses of these growth conditions, such as substrates species, substrate temperature, and oxygen pressure are necessary. For chemical vapor deposition, the concentration of precursors is also an important parameter that is related to the stoichiometry of the deposited film.

### 4.1. Physical Vapor Deposition (Sputter and PLD)

A target is shot by pulsed laser or plasma in the methods of PLD or sputter, respectively. Both methods often cause oxygen deficiency and polycrystalline structures of thin films. The standard diffraction data for the ZnGa_2_O_4_ film is considered in this review article by the Joint Committee on Powder Diffraction Standards card file 38–1240. Growth conditions based on different substrates, substrate temperature, oxygen pressure, and annealing temperature will be discussed here. The process parameters for sputter and PLD are listed in Table 3 and Table 4, respectively.

#### 4.1.1. Effect of Substrate Materials

**Figure 6:**
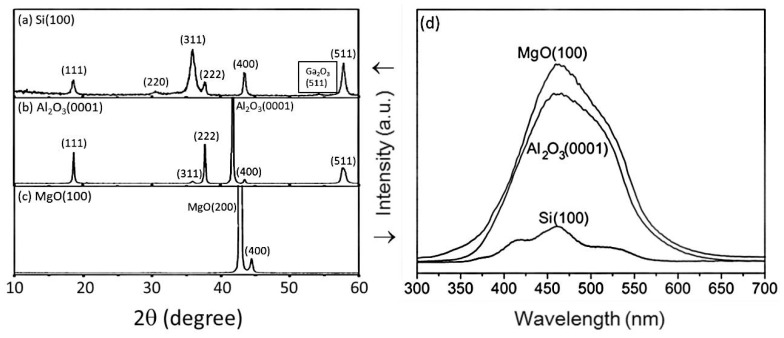
XRD pattern of ZnGa_2_O_4_ thin film deposited on: (**a**) Si(100) substrate; (**b**) Al_2_O_3_(0001) substrate; (**c**) MgO(100) substrate; (**d**) room temperature PL spectra of ZnGa_2_O_4_ films grown on these substrates (substrate temperature: 550 °C and oxygen pressure: 100 mTorr by PLD) [80] (with copyright permission from Elsevier, 2020).

There are some popular substrates such as silicon Si(100), sapphire(0001), and MgO(100). Polycrystalline films were obtained on Si(100) substrates [80], while single-phase crystalline films could be obtained on MgO(100) substrates when certain values of oxygen pressure and temperature were applied. Luminescence characteristics of ZnGa_2_O_4_ thin film phosphors on various substrates were investigated by pulsed laser deposition, as shown in Figure 6. The XRD pattern of the ZnGa_2_O_4_ thin film on Si(100) substrate is shown in Figure 6a. For the standard powder diffraction on Si(100) substrate, (311) is found as the main peak, and (222) is the preferred orientation for ZnGa_2_O_4_ thin film [33,72].

The XRD pattern of the ZnGa_2_O_4_ thin film on the sapphire Al_2_O_3_(0001) substrate is shown in Figure 6b. This XRD pattern shows that the films are perfectly crystallized with mixed orientations, primarily (111) [71]. Peak (111) is found as the lattice-matched orientation with the sapphire substrate [71,80]. Secondary orientations are observed as (311) and (511) peaks [71,80]. The crystalline orientation of ZnGa_2_O_4_ thin film on MgO(100) substrate is (400), as shown in Figure 6c. The substrate temperature and oxygen partial pressure should be 600 °C and 100 mTorr or higher, respectively for epitaxial films with ZnGa_2_O_4_/ZnO (50%/50%) target to obtain the stoichiometric single phase [79].

For these same conditions, three different kinds of substrates, Si(100), Al_2_O_3_(0001), and MgO(100), were taken into consideration for the deposition of ZnGa_2_O_4_ film and compared by testing their PL spectrums, as shown in Figure 6d. From Figure 6d, one can observe that the film deposited on MgO(100) has obtained the best crystallinity among all substrates. The ZnGa_2_O_4_ film deposited on the Si(100) substrate has the lowest crystallinity, which agrees with the randomly oriented reflections of the ZnGa_2_O_4_ thin film on the Si(100) substrate [71,80]. The PL spectrum as shown in Figure 6d revealed that luminescence brightness depends on the substrate material.

**Figure 7:**
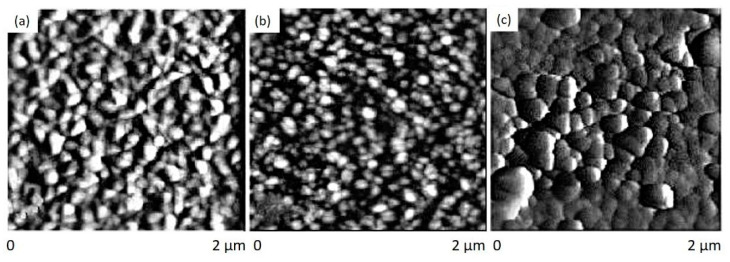
Atomic force microscope images of the ZnGa_2_O_4_ films grown on (**a**) Si(100), (**b**) Al_2_O_3_(0001) and (**c**) MgO(100) substrates at substrate temperature of 550 °C and oxygen pressure of 100 mTorr [80] (with copyright permission from Elsevier, 2020).

The lattice mismatch for ZnGa_2_O_4_ thin film with MgO(100), Al_2_O_3_(0001), and Si(100) substrates are 1%, 6%, and 12%, respectively. Hence, the deposition of ZnGa_2_O_4_ film on different substrates revealed that ZnGa_2_O_4_ films possessed different crystallinity and morphology. A comparison between epitaxial and polycrystalline films has been performed in order to investigate the influence of granularity on luminescence. The grain shapes showed different morphology such as mixed of several types, pair rod type and square types for ZnGa_2_O_4_ films on Si(100), Al_2_O_3_(0001), and MgO(100), respectively, as shown in Figure 7a–c. Large grain size and surface roughness were observed with the films grown on MgO(100) substrate [71].

**Figure 8:**
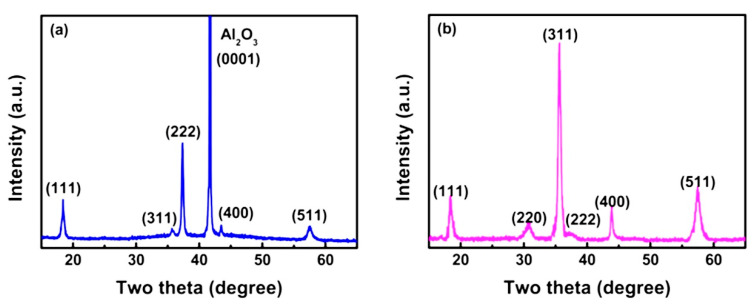
XRD patterns of ZnGa_2_O_4_ films grown on: (**a**) sapphire(0001) and (**b**) Si(100) substrates [78].

The PLD method has some disadvantages, such as large clusters that often occur during the ablation process, which brings non-homogeneity and increased surface roughness. However, RF magnetron sputtering is an effective and low-cost method to deposit thin films that can achieve good uniformity over a large area. Wang et al. investigated the structural and photoluminescence characteristics of ZnGa_2_O_4_ thin films using RF magnetron sputtering on sapphire and Si(100) substrate [78]. The structural characteristics of ZnGa_2_O_4_ thin films deposited on sapphire and Si(100) substrates are shown in Figure 8a,b. The structural characteristics exhibited that the ZnGa_2_O_4_ films possessed polycrystalline nature of the films when deposited on the sapphire substrate and found randomly oriented, primarily with the (111), (222) and (511) planes parallel to the substrate surface. However, the deposition of the ZnGa_2_O_4_ film on the Si(100) substrate exhibited that the film was polycrystalline and randomly oriented with (311)- and (020)-planes.

**Figure 9:**
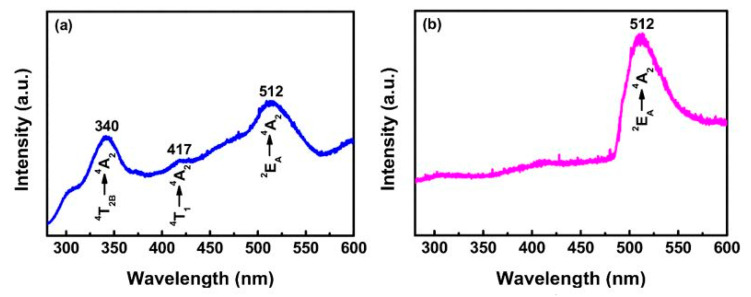
Room temperature PL spectra of the ZnGa_2_O_4_ films deposited on: (**a**) sapphire(0001) and (**b**) Si(100) substrates [78].

The photoluminescence spectra have been shown in Figure 9a,b. The strongest emission peaks of the ZnGa_2_O_4_ film were deposited on the sapphire substrate, peaking at 340 and 512 nm, which can be ascribed to ^4^T_2B_ → ^4^T_2A_ transitions. However, the film deposited on the Si(100) substrate emitted only an intrinsic green emission, which corresponds to ^2^E_A_ → ^4^T_2A_ transition with a peak at 512 nm in the PL spectrum. These differing strongest and dominant emissions could be attributed to the substitution of Ga^3^^+^ ions at Zn^2^^+^ sites during the high-temperature deposition process and the nature of the substrate, which proved that the substrate material influenced the dominant optical transitions. The structural and photoluminescence properties of sputter-deposited ZnGa_2_O_4_ thin film on the sapphire substrate indicated that the sapphire substrate is suitable for the growth of polycrystalline, high-quality ZnGa_2_O_4_ thin film by RF magnetron sputtering.

#### 4.1.2. Effects of Substrate Temperature and Oxygen Pressure

**Figure 10:**
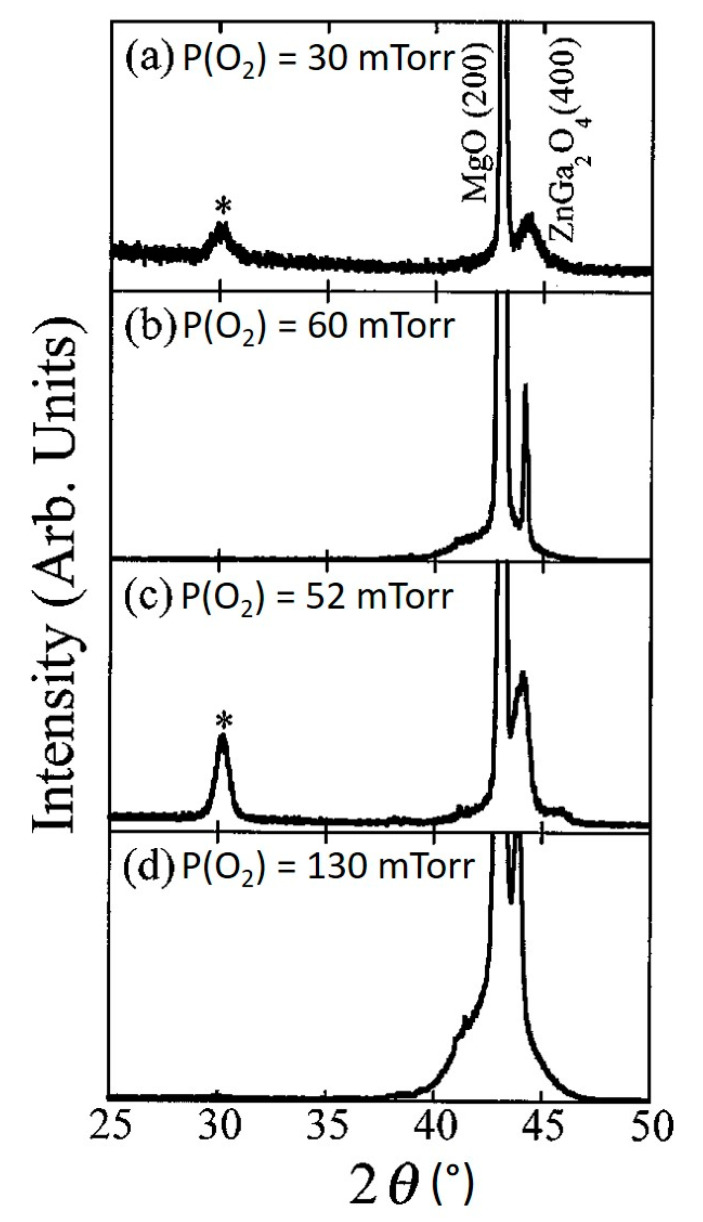
X-ray scans for ZnGa_2_O_4_ films deposited at 650 °C on MgO(100) substrates under P(O_2_) of (**a**) 30 and (**b**) 60 mTorr with total pressure fixed at 60 mTorr; and at 700 °C on MgO(100) substrates under P(O_2_) of (**c**) 52 and (**d**) 130 mTorr with total pressure fixed at 130 mTorr using an area portion of ZnGa_2_O_4_/ZnO of 50%/50% target [79] (with copyright permission from AIP Publishing, 2020).

Oxygen plays an important role in the growth of ZnGa_2_O_4_ thin films. If oxygen gas is insufficient during the growth, oxygen vacancies will take place in the deposited film and Zn has more possibility to diffuse out. Thus, the stoichiometry of the film will not be the same and the crystallinity will be poor. Figure 10a–d shows the structural characteristics of the ZnGa_2_O_4_ film deposited at a different substrate temperatures of 650 and 700 °C, as well as with different oxygen pressures of 60 and 100 mTorr [79]. The films deposited with low oxygen temperature show a (400) ZnGa_2_O_4_ peak along with another diffraction peak at 30° which corresponds to the Ga_2_O_3_ phase. The introduction of additional oxygen will produce more Zn-O molecules through gas-phase collisions of Zn species with oxygen. Increasing partial oxygen pressure P(O_2_) decreases the Zn loss in the ZnGa_2_O_4_ film and a near stoichiometric single phase can be found for epitaxial ZnGa_2_O_4_ films when the substrate temperature of 600 °C or higher and P(O_2_) of 60 mTorr or higher must be kept fixed while using the ZnGa_2_O_4_/ZnO target (50%/50%).

**Figure 11:**
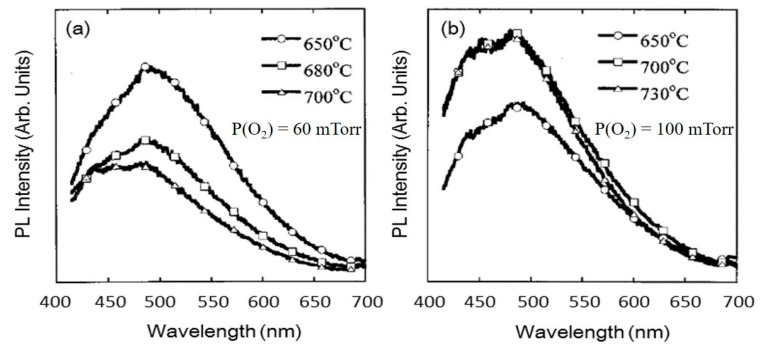
PL emission spectra of ZnGa_2_O_4_ films on MgO(100) substrates using an area portion of ZnGa_2_O_4_/ZnO of 50%/50% target with various substrate temperatures under total P(O_2_) of (**a**) 60 and (**b**) 100 mTorr [79] (with copyright permission from AIP Publishing, 2020).

The effect of substrate temperature along with partial oxygen pressure on the PL emission spectra for the ZnGa_2_O_4_ films grown on MgO(100) substrates using the ZnGa_2_O_4_/ZnO (50%/50%) target is shown in Figure 11. It is evident from Figure 11a that increasing the substrate temperature reduced the PL intensity of the deposited film when P(O_2_) of 60 mTorr is kept fixed during the growth. However, if P(O_2_) of 100 mTorr or higher is kept fixed during the growth then PL intensity is found to increase with the increase in substrate temperature as shown in Figure 11b. These results indicated that substrate temperature and oxygen pressure are crucial parameters that affect the crystallinity and luminescence properties.

**Figure 12:**
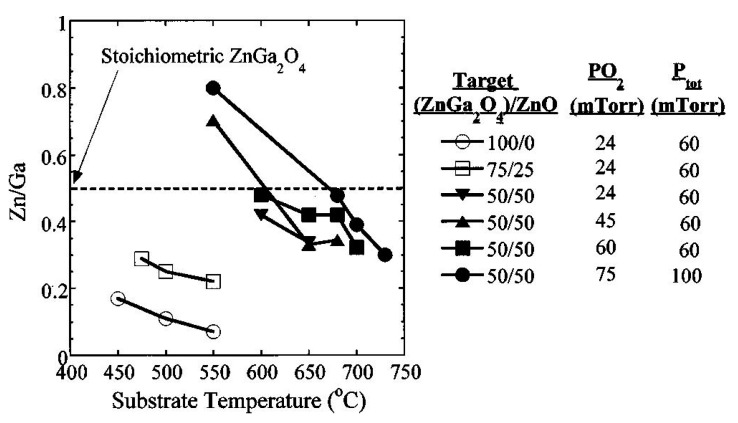
Variation of Zn/Ga ratio in ZnGa_2_O_4_ films with various types of ZnGa_2_O_4_/ZnO targets and substrate temperatures [79] (with copyright permission from AIP Publishing, 2020).

Zinc has higher vapor pressure than gallium, which indicates that zinc has more possibility to evaporate than gallium. Increasing the substrate temperature leads to evaporate the zinc content and therefore it is expected that the Ga_2_O_3_ phase can a place in the ZnGa_2_O_4_ thin film. To overcome the loss of Zn, some researchers used the Zn_0.97_Ga_0.03_O target [81]. ZnGa_2_O_4_ exhibits a strong blue emission and the regular Ga-O octahedron is the reason behind this self-activated blue emission characteristic of ZnGa_2_O_4_ while distorted Ga-O octahedron which originates from the oxygen and zinc vacancies is the reason behind the ultraviolet emission [82]. Therefore, the shift in the PL spectrum to a shorter wavelength with decreasing Zn/Ga ratio takes place [79]. If the oxygen pressure is not kept fixed at 100 mTorr or higher than 100 mTorr, then increasing the substrate temperature will lead to lower PL intensity and poor crystallinity, as shown in Figure 11a. As a result, a decrease in Zn/Ga ratio can be observed in Figure 12 [79]. This Zn/Ga ratio for different oxygen partial pressures along with different proportions of the ZnGa_2_O_4_/ZnO target as a function of substrate temperature is shown in Figure 12. It is clear from Figure 12 that if the oxygen pressure is kept fixed at 100 mTorr or higher and applied during the growth, then the increasing temperature results in an enhanced PL intensity, but the decrease from 0.47 to 0.30 in the ratio of Zn/Ga takes place with a ZnGa_2_O_4_/ZnO (50%/50%) target.

**Figure 13:**
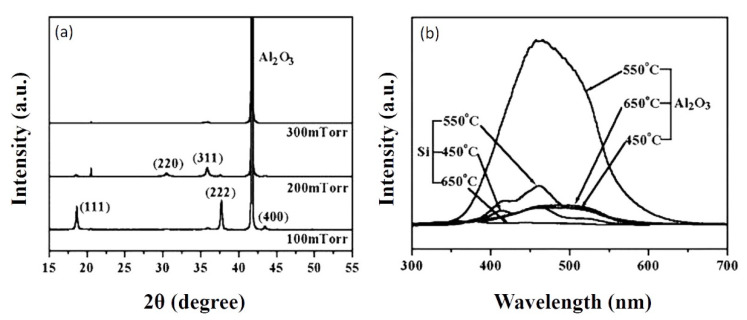
(**a**) XRD patterns of ZnGa_2_O_4_ films grown on Al_2_O_3_(0001) substrate at different oxygen growth pressures, (**b**) room-temperature PL spectra of ZnGa_2_O_4_ films grown on Si(100) and Al_2_O_3_(0001) substrates at different substrate temperatures of 450, 550, 650 °C (100 mTorr oxygen pressure) [71] (with copyright permission from Elsevier, 2020).

The effect of substrate temperature and oxygen pressure depends on the substrate material. We have discussed the effect of substrate temperature and oxygen pressure for ZnGa_2_O_4_ thin film on MgO(100) substrate where increasing partial pressure improves the crystallinity. Now, we will discuss the effect of substrate temperature and oxygen pressure on sapphire(0001) substrate where increasing oxygen pressure does not improve crystallinity every time [71]. The structural characteristics, as shown in Figure 13a, revealed that the film grown at an oxygen pressure of 100 mTorr was perfectly crystallized with the preferred orientation (111) on sapphire(0001) substrate. Increased oxygen pressure from 100 to 300 mTorr led to changes in the crystalline orientation of ZnGa_2_O_4_ films from (111) to polycrystalline orientation (220) and (311) [71]. Similarly, increasing substrate temperature does not always reduce PL intensity. Bae et al. reported that initially the PL intensity was increased with the increasing substrate temperature below 550 °C and later it decreased when the substrate temperature was increased above 550 °C, as shown in Figure 13b [71].

Hence, the substrate temperature of 550 °C and oxygen pressure of 100 mTorr is found suitable to be employed as the optimized growth parameters for obtaining the crystalline ZnGa_2_O_4_ films for photoluminescence investigations.

#### 4.1.3. Effect of Annealing Temperature

**Figure 14:**
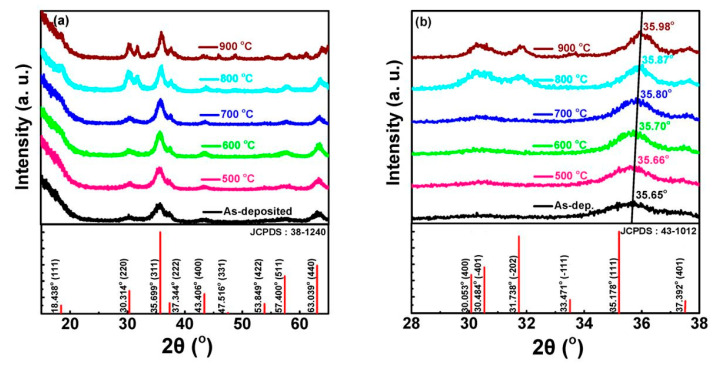
(**a**) XRD spectra of ZnGa_2_O_4_ films at different annealing temperatures; (**b**) XRD spectra of ZnGa_2_O_4_ showing a limited 2 Theta range (28° to 38°) [74].

Annealing is a popular method to enhance the crystallinity of ZnGa_2_O_4_ thin films, although this method is accompanied by Zn depletion and oxygen deficiencies in the films, thus resulting in the deformation of the ZnGa_2_O_4_ compound into its constituents (ZnO and Ga_2_O_3_). Wang et al. reported the influence of annealing temperature on the ZnGa_2_O_4_ thin films on a c-plane sapphire substrate using RF magnetron sputtering [74]. The films were deposited at a substrate temperature of 400 °C and annealing temperature was increased from 500 to 900 °C in order to improve the crystallinity of the films. Figure 14 shows the structural characteristics of ZnGa_2_O_4_ thin films deposited on the sapphire substrate. The intensity of the diffraction peak (311) was found to be increased with increasing the annealing temperature. As the annealing temperature increased above 800 °C, diffraction peaks of Ga_2_O_3_ (-401) and (-202) were observed from the phase separation of ZnGa_2_O_4_.

**Figure 15:**
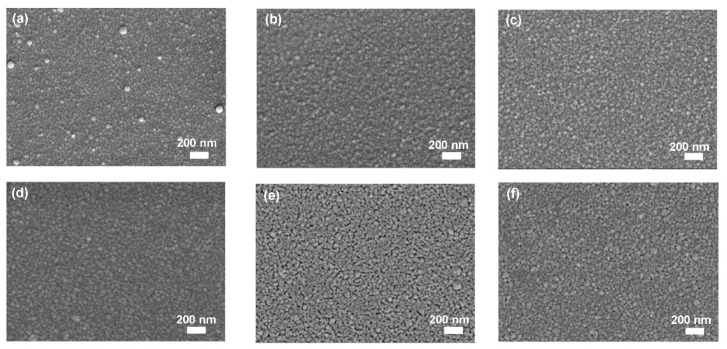
SEM micrographs of ZnGa_2_O_4_ thin film samples at different annealing temperatures of (**a**) as-deposited, (**b**) 500, (**c**) 600, (**d**) 700, (**e**) 800 and (**f**) 900 °C [74].

Figure 15a–f shows plane-view SEM images of the ZnGa_2_O_4_ films at different annealing temperatures. Surface morphologies of these ZnGa_2_O_4_ films show a very similar column structure. Annealing temperature increases the crystallite size which is due to the regrowth and coalescence during thermal treatment. High annealing temperature provides sufficient driving force to improve the mobility of the atoms and improves the film crystallinity. Annealing temperature improves the crystallinity of the films.

**Figure 16:**
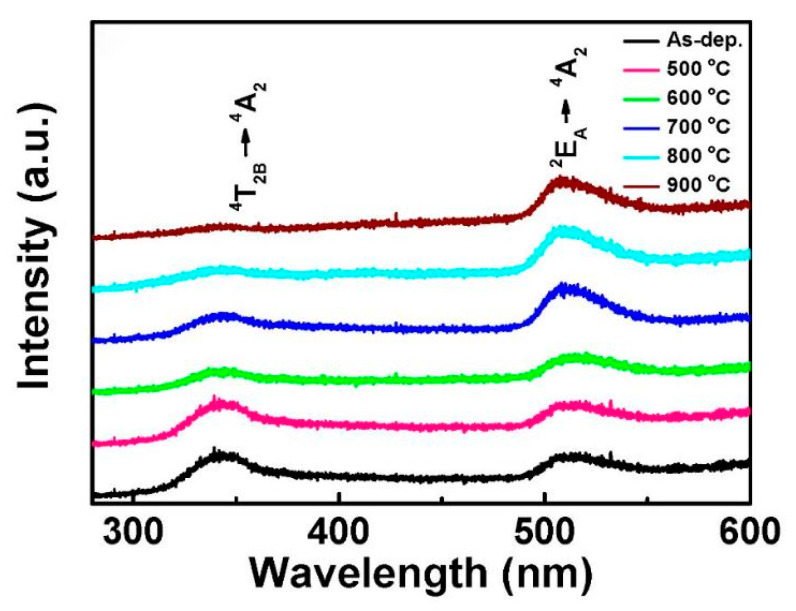
Photoluminescence spectra of ZnGa_2_O_4_ films annealed at different temperatures [74].

Figure 16 shows the photoluminescence emission spectra of ZnGa_2_O_4_ thin films at various annealing temperatures. It was observed that when ZnGa_2_O_4_ film annealed at higher temperature possess a higher luminescence intensity. For photoluminescence spectra, each annealed film exhibited broad-band emission extending from 300 to 600 nm, the emission peaks were located at 340 and 520 nm. The emission peak centered at 340 nm can be attributed to the ^4^T_2B_ → ^4^T_2A_ transition while the ^2^E_A_ → ^4^T_2A_ transition is responsible for the emission peak centered at 520 nm. UV band emission is related to the excited excess Ga^3^^+^ ions of the Ga-O group.

### 4.2. Chemical Vapor Deposition (Mist CVD and MOCVD)

Polycrystalline ZnGa_2_O_4_ films have been investigated for phosphor applications including luminescence characteristics in the previous decades; however, single-crystalline ZnGa_2_O_4_ films have been investigated recently based on optical properties such as transparency and cathodoluminescence. Among the various growth methods aforementioned above, metal-organic chemical vapor deposition (MOCVD) is a beneficial technology for obtaining high-quality single-crystalline films. High deposition rate, conformal mapping over a complex structure, and large area uniformity, which are helpful to improve the characteristics of the deposited films, show advantages of MOCVD. Precise control of the substrate temperature and oxygen partial pressure is necessary, in order to get near stoichiometric single crystalline films when deposited by pulsed laser deposition [83] because of the highly volatile nature of Zn. Hence, ZnGa_2_O_4_ thin films deposited by mist chemical vapor deposition (mist CVD) and metal organic chemical vapor deposition (MOCVD) have been investigated. The process parameters of the above two kinds of CVD methods are listed in Table 5. Controlling the Zn/Ga ratio by varying amounts of precursors is the main issue in depositing ZnGa_2_O_4_ thin films by CVD methods. Therefore, special focus is given on the relation between the Zn/Ga ratio and the amount of precursor used in the deposition of ZnGa_2_O_4_ thin films. Their CL properties will be also discussed here.

#### 4.2.1. Effect of Zn/Ga Precursor Ratio

The Zn/Ga precursor ratio plays an important role in obtaining the near stoichiometric single-crystalline ZnGa_2_O_4_ films. When the less concentration of the Zn precursor is taken into account, the Ga_2_O_3_ phase has more possibilities to appear in the deposited ZnGa_2_O_4_ films. In contrast, if the excess concentration of the Zn precursor is taken into account, then the ZnO phase also has the possibility to appear in the deposited ZnGa_2_O_4_ films [83]. The sublimation rate of Zn is high and keeping this fact in account, the precursor of Zn should be taken more than the precursor of Ga to form ZnGa_2_O_4_ in stoichiometry. Horng et al. investigated the ZnGa_2_O_4_ films grown on c-plane sapphire substrates by MOCVD where diethylzinc (DEZn), triethylgallium (TEGa), and oxygen were used as the precursors [76]. An increase in the flow rate of DEZn resulted in the transformation from the Ga_2_O_3_ phase to the ZnGa_2_O_4_ phase.

**Figure 17:**
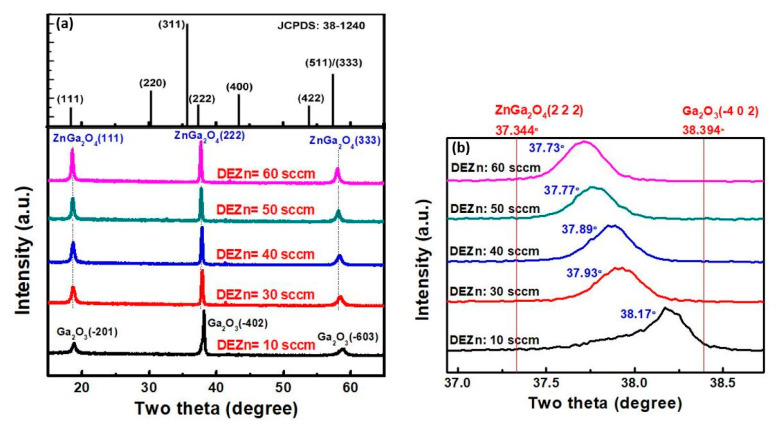
(**a**) XRD patterns of ZnGa_2_O_4_ films prepared at various DEZn flow rates of 10−60 sccm and (**b**) XRD patterns of ZnGa_2_O_4_ films prepared at various DEZn flow rates of 10−60 sccm with the limited 2θ range from 36.9° to 38.7° [76] (with copyright permission from American Chemical Society, 2020). Joint Committee on Powder Diffraction Standards (JCPDS) data of the normal spinel ZnGa_2_O_4_ (card no. 38-1240) is given for reference.

Horng et al. reported that increasing the DEZn flow rate results in the transformation from the *β*-Ga_2_O_3_ to the ZnGa_2_O_4_ phase, and that the diffraction peak of ZnGa_2_O_4_ shifts to a lower angle, as shown in Figure 17a,b [76]. This shift in peak position can be attributed to the inclusion of Zn^2+^ in the gallium oxide structure where the atomic radius of Zn^2+^ and Ga^3+^ is 0.74 and 0.63 Å, respectively. The elemental compositions for different flow rates of DEZn are listed in Table 6. The Ga/Zn in the film is approaching 2, which is the standard stoichiometric number of ZnGa_2_O_4_ with the increasing flow rate of DEZn. This phenomenon indicates that the higher flow rate of DEZn leads to the ZnGa_2_O_4_ phase, and that no other impurity phase is observed.

**Figure 18:**
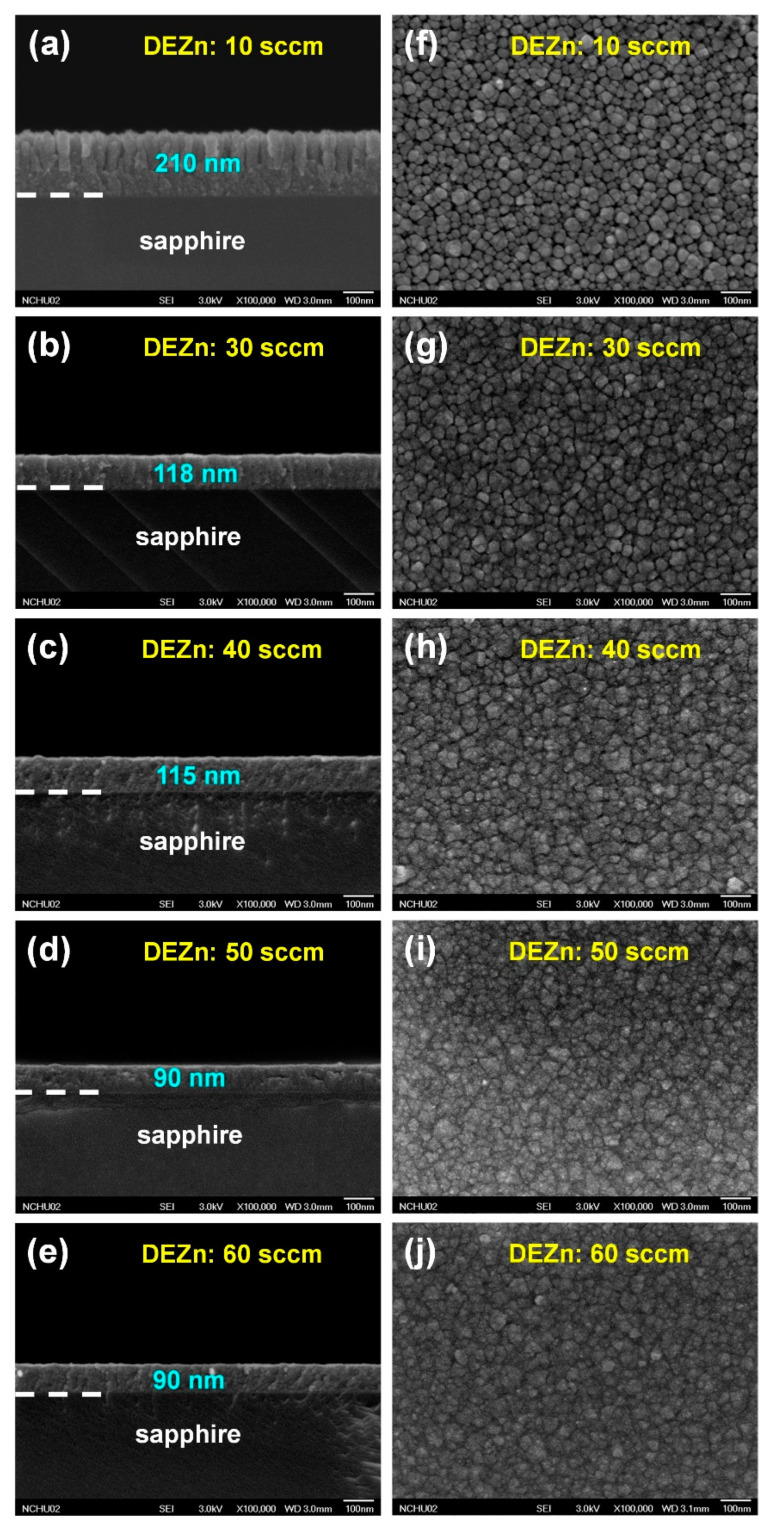
(**a**–**j**) Cross-sectional and corresponding plan-view images of SEM for ZnGa_2_O_4_ films deposited at the DEZn flow rates of 10, 30, 40, 50, and 60 sccm [76] (with copyright permission from American Chemical Society, 2020).

Figure 18a–e shows the cross-sectional SEM images and Figure 18f–j shows the plane-view SEM images of ZnGa_2_O_4_ films deposited at DEZn flow rates of 10, 30, 40, 50, and 60 sccm [76]. Figure 18f–j shows that the surface morphologies of these ZnGa_2_O_4_ films are very similar to each other, however Figure 18a–e reveals from the cross-sectional SEM images that there are differences in the surface morphology between the ZnGa_2_O_4_ films deposited at DEZn flow rates of 10 and 30–60 sccm. The columnar structure has been found for the film deposited with DEZn flow rates of 10 sccm, however a film-type structure has been found when the DEZn flow rate was increased higher than 30 sccm. The DEZn flow rate of 10 sccm had a doping effect on the Ga_2_O_3_ structure, while the addition of Zn added into the ZnGa_2_O_4_ films prepared at DEZn flow rates of 30–60 sccm reacted with Ga and O atoms to form the ZnGa_2_O_4_ structure.

The value of O/(Zn + Ga) for pure Ga_2_O_3_ is 1.5, while pure ZnGa_2_O_4_ is 1.33. It is clear from Table 6 that the amount of oxygen vacancies in the ZnGa_2_O_4_ film grown at the DEZn flow rate of 10 sccm is very large compared to pure Ga_2_O_3_. Compared to pure ZnGa_2_O_4_, the amounts of oxygen vacancies in the ZnGa_2_O_4_ films prepared at 30 and 40 sccm are still large. However, ZnGa_2_O_4_ films prepared at 50 and 60 sccm possess fewer oxygen vacancies than the other films, because the values of O/(Zn + Ga) for these two films are 1.29 and 1.20, which are close to that of ZnGa_2_O_4_: 1.33.

**Figure 19:**
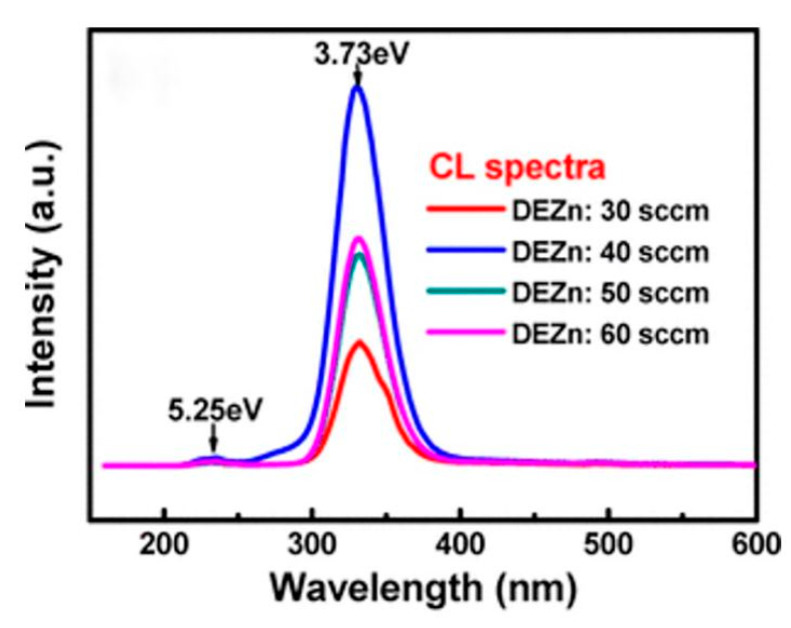
CL spectra of ZnGa_2_O_4_ films prepared at the DEZn flow rates of 30−60 sccm [76] (with copyright permission from American Chemical Society, 2020).

Figure 19 shows the cathodoluminescence properties of ZnGa_2_O_4_ films prepared at DEZn flow rates of 30–60 sccm. Luminescence peak at a wavelength of 332 nm was found in all films, and its corresponding bandgap value was evaluated to be 3.73 eV. The strong emission band was found at 332 nm which is due to a radiative carrier transition from the donor level to the valence band. In addition to this strong emission band at 332 nm, two weak luminescence peaks at wavelengths of 236 and 499 nm can be also found in all films, and their bandgap values are 5.25 and 2.48 eV, respectively. Incorporation of Zn into Ga_2_O_3_ to form ZnGa_2_O_4_ would result in a donor–acceptor pair transition. Therefore, the intrinsic green emission band (499 nm) can be efficiently suppressed. The luminescence peak at 236 nm could be attributed to the transition of electrons from the conduction band to the valence band, and its bandgap value was 5.25 eV, which is very close to the theoretical bandgap value of ZnGa_2_O_4_ (5.2 eV). The two distinct luminescence peaks centered at 236 and 332 nm revealed that these ZnGa_2_O_4_ films are highly promising for short-wavelength applications [76].

#### 4.2.2. Effect of Substrate Temperature

**Figure 20:**
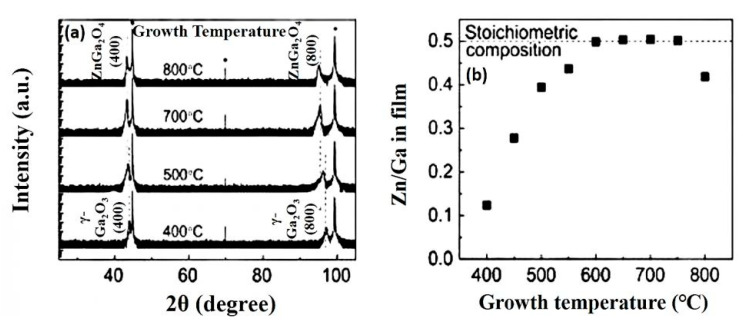
(**a**) Out-of-plane XRD patterns of the films grown at various temperatures using a precursor solution with [Zn]/[Ga] = 1. Asterisks (*) refer to the substrate peaks. Dashed lines indicate peak angles of ZnGa_2_O_4_ and γ-Ga_2_O_3_ bulks. (**b**) Growth temperature dependence of Zn/Ga in the films as obtained in deposited films [83] (with copyright permission from Elsevier, 2020).

The substrate temperature/growth temperature affects the crystallinity as well as the Zn/Ga ratio in the deposited ZnGa_2_O_4_ films. Oshima et al. deposited ZnGa_2_O_4_ thin films on MgAl_2_O_4_(100) substrates with a fixed precursor ratio [Zn]/[Ga] of 1 [83]. [Zn]/[Ga] represents the ratio of precursors used in the deposition of ZnGa_2_O_4_ films while Zn/Ga represents the stoichiometry obtained in the deposited ZnGa_2_O_4_ films. When the growth temperature was fixed at 400 °C, then reflections from the metastable γ-phase Ga_2_O_3_ have been observed as shown in Figure 20a. As the growth temperature increased, the ZnGa_2_O_4_ phase was found to be dominant. Stoichiometric composition of ZnGa_2_O_4_ was obtained when the growth temperature was increased from 600 to 750 °C as shown in Figure 20b. If the growth temperature exceeds 750 °C, then the sublimation of the ZnO phase is significant, which leads to low γ-Ga_2_O_3_ phase formation in the deposited ZnGa_2_O_4_ film.

**Figure 21:**
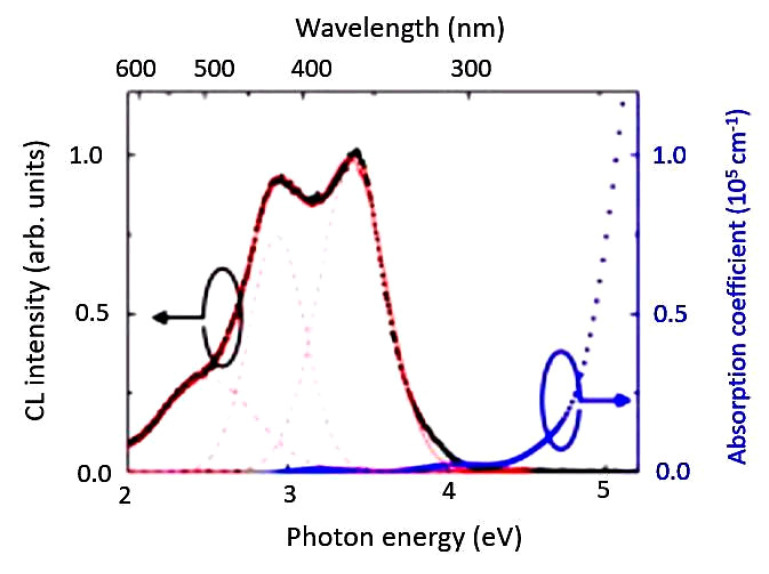
Cathodoluminescence (CL) and absorption spectra of the ZnGa_2_O_4_ film. The CL spectrum is deconvoluted with three Gaussian curves centered at 2.5 eV (495 nm), 2.9 eV (428 nm), and 3.4 eV (365 nm) (dashed lines). The experimental data (■) is superimposed by the best-fit curve (solid line) [83] (with copyright permission from Elsevier, 2020).

Room temperature cathodoluminescence and absorption spectra of the film are shown in Figure 21. Three distinct emission bands peaked at 495, 428 and 365 nm have been found and their photon energy is 2.5, 2.9, and 3.4 eV, respectively. The stronger emission at 365 nm (3.4 eV) and 428 nm (2.9 eV) can be assigned as bulk properties which originated from charge transfers between Ga^3+^ and octahedrally coordinated O^2−^ with regular and distorted octahedral sites, respectively. The weaker emission at 495 nm (2.5 eV) is rarely observed in ZnGa_2_O_4_, which appeared from either defects or impurities. Single-crystalline ZnGa_2_O_4_ films are epitaxially grown on MgAl_2_O_4_ substrates by mist CVD. Their work has contributed to future studies on ZnGa_2_O_4_-spinel-based oxide semiconductors towards potential applications.

## 5. Applications of ZnGa_2_O_4_

ZnGa_2_O_4_ is a wide-bandgap, conductive, chemically and thermally stable semiconductor. ZnGa_2_O_4_ has received significant attention these days because it has potential applications in missile threat attention, solar-blind ultraviolet (UV) photodetectors (PDs), photocatalyst, UV radiation monitor in the environment, and lithography alignment [84,85]. UV photodetectors fabricated by using ZnGa_2_O_4_ thin films are discussed here in detail. The other applications such as phosphors and gas sensors will also be mentioned.

### 5.1. Deep-Ultraviolet Photodetectors

Optoelectronics devices can be operated in the visible, infrared, or ultraviolet spectral region. Photodetectors are one of the optoelectronic devices that are used in various fields, such as flame monitoring, missile warning, environmental monitoring, biological and chemical analysis, optical communication, space research and so on [86]. With a bandgap of 4.4–5.0 eV, ZnGa_2_O_4_ has potential to detect from violet to the near-UV region of the spectrum [87], even to deep-ultraviolet (DUV). An excellent DUV photodetector consists of properties such as low dark current, high responsivity, high quantum efficiency, fast response time, and high DUV-visible rejection ratio [85].

Metal-semiconductor-metal (MSM) DUV photodetectors possess low dark current as well as excellent photoelectric characteristics [85]. When the active area is the same, the implementation of interdigitated electrodes in MSM photodetectors has resulted in a significant increase in bandwidth compared with standard conventional PIN photodiodes [88]. MSM photodetectors possess low capacitance and have the potential for large-area detectors [89]. Therefore, MSM ZnGa_2_O_4_ photodetectors in Reference [20,85,90,91] are taken into consideration. Their process parameters and optimized performances are listed in Table 7 and Table 8, respectively.

#### 5.1.1. MSM DUV Photo-Detecting Mechanism

**Figure 22:**
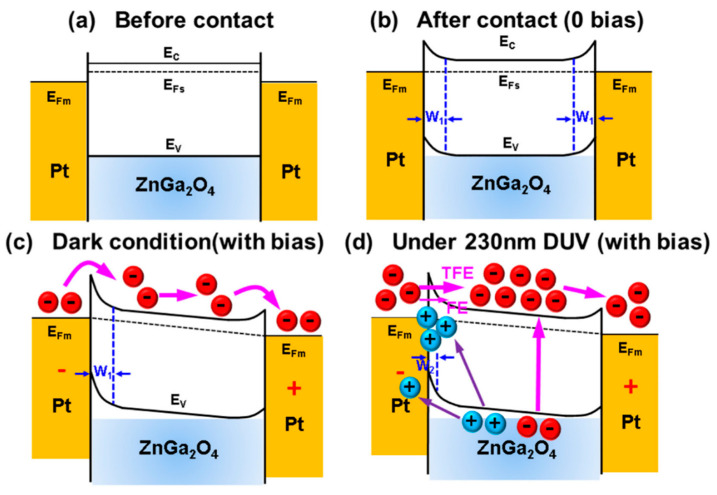
Energy-band diagram of metal-semiconductor-metal (MSM) structure (**a**) before metal contact, (**b**) after metal contact, (**c**) in dark conditions, and (**d**) under deep-ultraviolet (DUV) illumination [85] (with copyright permission from Elsevier, 2020).

To investigate the back-to-back MSM structure, a schematic energy band diagram has been taken from Reference [85] to explain the mechanism of the metal-semiconductor (MS) junction and the carrier transport phenomenon between them. Platinum (Pt) was used as the metal and an n-type ZnGa_2_O_4_ was used as the semiconductor, to make the metal-semiconductor junction as shown in Figure 22.

Figure 22a shows the schematic energy band diagram of isolation between the metal and semiconductor with each other. When the metal and semiconductor are brought into contact with each other, then Fermi levels of the metal and semiconductor lined up and induced the band bending which emerged the Schottky barrier at the MS junction, as shown in Figure 22b. The work function of the platinum metal is in the range of 5.12–5.93 eV while work function of n-type ZnGa_2_O_4_ is 3.4 eV. The work function is defined as the energy difference between the Fermi level and the vacuum level. Note, the higher work function of the metal than of the semiconductor leads to the Schottky barrier at the MS junction. A conventional space charge region depletion region (W_1_) took place at the MS junction because of shallow ionized donors, and this depletion region was responsible for blocking the current flow from semiconductor to metal. Bartolomeo explained a clear and wonderful interpretation related to the mechanism of the metal-semiconductor (MS) junction, as in Reference [93].

Schottky barriers were influenced by forward or reverse bias in the dark condition, and accordingly, the barrier height decreased or increased separately, as shown in Figure 22c. In reverse bias, leakage current flows into the photodetector in the absence of DUV, which is known as dark current. Several free electron-hole pairs are generated when DUV light is incident on the semiconductor as shown in Figure 22d. When the reverse bias is applied, the photogenerated holes were mostly trapped in hole traps. The redistribution of the space charge that appeared in the left MS junction resulted in an increase in the positive charge density in the depletion region which causes the Schottky barrier to reduce in width to W_2_. Moreover, the accumulated positive holes can easily attract electrons from the cathode into the semiconductor. The narrowing of the depletion region allowed the electrons to tunnel easily in the left MS, resulting in the enhancement of photoconductive gain under DUV illumination. In addition, the band model of the Schottky barrier with the forward and reverse bias has been clearly described and proposed by Bartolomeo et al., which can be found in Reference [92].

#### 5.1.2. Dark Current and Photocurrent

With the application of forward and reverse bias, the MSM structure may have an almost identical I-V curve. The current increases with the applied bias voltage due to the increased carrier drift velocity [91]. With no light illumination on a semiconductor, a current exists in MSM PDs called dark current (I_dark_). When light is incident on the semiconductor between the electrodes, it generates electrons and holes which are collected by the electric field and thus produces photocurrent (I_ph_). Some bias is applied to the electrodes during the operation of photocurrent.

For better photodetector performance, the ratio of I_ph_/I_dark_ should be as large as possible. Tsai et al. found that the dark current of ZnGa_2_O_4_ MSM PD fabricated with as-deposited film exhibited the I_ph_/I_dark_ of one order (~10^1^ order) of magnitude, while ZnGa_2_O_4_ MSM PD fabricated with post-annealed ZnGa_2_O_4_ film at a temperature of 800 °C exhibited the ratio I_ph_/I_dark_ of 8 orders (~10^8^ order) [91]. The reason behind the reduction of the dark current is post-annealing, which reduced the defects and improved the ZnGa_2_O_4_ MSM PDs performance.

In addition to the annealing method, increase in the partial oxygen pressure also changes the concentration of oxygen vacancies [20], resulting in less oxygen vacancies or less carriers in the ZnGa_2_O_4_ thin films. The concentration of oxygen vacancies directly affects photocurrent and dark current because the concentration of carriers is directly proportional to the current. Therefore, both dark current and photocurrent are decreased due to an increase in partial oxygen pressure. However, the ratio of I_ph_/I_dark_ did not reach a higher value with increasing partial oxygen pressure [20].

**Figure 23:**
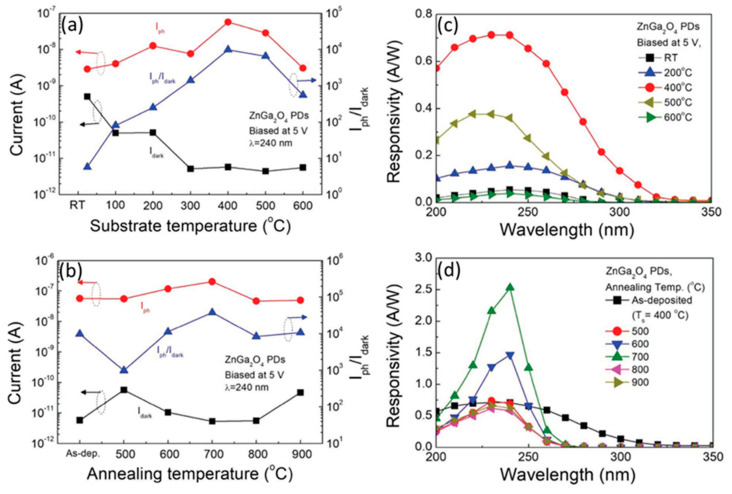
The photocurrent (I_ph_, red circles), dark current (I_dark_, black squares), and I_ph_/I_dark_ current ratio (blue triangles) of the ZnGa_2_O_4_ photodetectors (PDs) as a function of (**a**) substrate temperatures (T_s_) from 25 to 600 °C and (**b**) annealing temperature (T_a_) from 500 to 900 °C, which were prepared under the incident light wavelength of 240 nm. The responsivities of ZnGa_2_O_4_ PDs as a function of wavelength with various (**c**) substrate temperature T_s_ and (**d**) annealing temperature T_a_ [90]. (with copyright permission from John Wiley and Sons, 2020).

Increasing substrate temperature also influences the ratio of I_ph_/I_dark_. Chen et al. reported in their work that when the substrate temperature (T_s_) was increased from room temperature (25 °C) to 300 °C, then the dark current decreased, and when T_s_ was increased from 300 to 600 °C, the I_dark_ showed a similar low value, as shown in Figure 23a [90]. Increasing substrate temperature could reduce defect states and as a result of this, the dark current was found to be reduced in the ZnGa_2_O_4_ PDs [90]. The substrate temperature of 400 °C is kept fixed as the optimized substrate temperature for the as-deposited ZnGa_2_O_4_ PDs. Figure 23b exhibits the I_ph,_ I_dark_, and I_ph_/I_dark_ for ZnGa_2_O_4_ PDs after annealing at different temperatures. 

#### 5.1.3. Spectral Response

The ratio of photocurrent per incident optical power defines the responsivity of a photodetector. Responsivity is represented by ‘R’ and given by:

R = I_ph_/P_inc_ (1)

where, P_inc_ is the incident optical power in Watts (W), I_ph_ is the photocurrent in amperes (A) [91]. Responsivity as a function of wavelength provides the spectral response of the photodetector. An excellent DUV photodetector possess high responsivity, fast response time, low dark current, and a high DUV-visible rejection ratio. Tsai et al. [85] increased the applied bias up to 20 V, investigated the DUV-visible rejection ratio of 3–4 orders (10^3^~10^4^), and reported the responsivity of 5.77 A W^−1^ under 230 nm DUV for the ZnGa_2_O_4_ PDs. The authors concluded that the Schottky barrier was responsible for this phenomenon [85]. Increasing the oxygen flow rate also results in enhancing the DUV-visible rejection ratio, but it is not feasible because less oxygen flow during sputtering can create more oxygen vacancies which could be excited by UV light [20].

In addition to the aforementioned methods above, post-annealing can transform the broad spectral response of the photodetector to sharp spectral response. Figure 23c exhibits that the highest responsivity of 0.71 A W^−1^ was achieved at T_s_ of 400 °C in which the responsivity peak exhibited the broad spectral response due to the defects present in the ZnGa_2_O_4_ PDs [90]. After post-annealing at a temperature of 700 °C, the responsivity reached 2.53 A W^−1^ from 0.71 A W^−1^ at 240 nm; then, the broad response was transformed to sharp spectral response, as shown in Figure 23d. Therefore, the performance of ZnGa_2_O_4_ PDs was improved by diminishing the number of defect states which improved the crystallinity and further the device performance. Hence, post-annealing treatment rearranged atoms and improved the characteristics of the film [90].

#### 5.1.4. Response Time of Photodetectors

The response time is determined by the two factors: the first one is the drift of the electrons and holes that are photogenerated in the depletion layer; the second is the diffusion of the electrons and holes that are photogenerated in the diffusion regions. Different applications require a particular response time of photodetectors [85]. Response time takes rise time and fall/decay time into consideration. Rise time is the time required for the output signal to change from 10% to 90% of the highest output value when the light is incident on the semiconductor, and the fall/decay time is the time required for the photodetector output level to change from 90% to 10% of the peak output level in the absence of incident light.

Chen et al. reported that the annealed ZnGa_2_O_4_ PD with an annealing temperature of 700 °C possesses a quasi-single-crystalline ZnGa_2_O_4_ structure, which exhibited its indirect bandgap property of spinel-ZnGa_2_O_4_ structure [90]. Some of the photogenerated carriers were excited to the higher energy state above the conduction band then returned to the conduction band by releasing energy, resulting in increasing its rise time. The decay time of annealed ZnGa_2_O_4_ film with an annealing temperature of 700 °C exhibited lower value than the as-deposited film [90].

In addition to the annealing method, increasing the partial oxygen pressure is another way to reduce oxygen vacancies and enhance the transient response of the photodetector. Huang et al. reported the effect of partial oxygen pressure by using three different samples A, B, and C, with partial oxygen pressures of 0%, 2%, and 4%, respectively [20]. The bias of 10 V and a UV irradiation of 260 nm were fixed during the measurements. The rise times of the samples A, B, and C were 13 s, 58 s, and 94 s, respectively. The samples with larger oxygen flows during sputtering possessed less oxygen vacancies, which resulted in less carriers and which further affects the time required for saturation to be longer. The decay times of samples A, B, and C were 2, 1, and 0.7 s, respectively, because carriers excited by light might be captured by oxygen vacancies which increased the recombination time [20]. Generally, a higher oxygen flow rate leads to longer rise time and shorter fall time.

### 5.2. Gas Sensors

Gas sensors are electronic devices that can be used to detect toxic, explosive, flammable gases and to measure their concentrations as well as oxygen depletion. Several types of gas sensors such as electrochemical, optical, and semiconductor gas sensors, etc., are in use at the industrial level—depending widely on the specific requirement. Each of them has its own advantages and disadvantages. The electrochemical gas sensor has a high sensitivity for low concentration, mainly used for low concentration biomedical sensors. However, its structure is more complicated, and its lifetime is limited by the internal electrolyte, which becomes the most fatal shortcoming of the electrochemical gas sensor. The most important elements of modern commercial production are having control over low running costs and practicality. Although the existing technology shows that the semiconductor gas sensor is difficult to sense at low concentration, its simple structure and low cost have considerable development potential.

Metal oxide semiconductors were studied well as their application in gas sensors. Sputtering and sol-gel were the key techniques to fabricate gas sensors from the decades, resulting in the amorphous or polycrystalline nature of thin films. Defects in the thin film were not easily controlled with these key techniques. Polycrystalline metal oxide semiconductors exhibit better sensing properties but suffer from poor selectivity and long response time [94].

The ZnO-Ga_2_O_3_ system possesses better sensing properties, hence zinc oxide and gallium oxide have been used as gas sensors for decades [95]. However, there are still relatively few publications about ZnGa_2_O_4_ gas sensors. For the first report about ZnGa_2_O_4_ gas sensors, Satyanarayana et al. [96] fabricated bulk ZnGa_2_O_4_-based gas sensors, which have high sensitivity and response rate to liquid-petroleum-gas (LPG). Jiao et al. [97] prepared ZnGa_2_O_4_ nanocrystals with a grain size of less than 10 nm by using an improved spray co-precipitation method. Chen et al. [98] reported that the ZnGa_2_O_4_/ZnO core-shell nanowires exhibit a linear relationship between sensitivity and nitrogen dioxide (NO_2_) concentration at temperatures of 25 and 250 °C.

ZnGa_2_O_4_ has shown significant performance in the detection of LPG and nitrogen dioxide gas (NO_2_) [99]. Gas sensing mechanism for the ZnGa_2_O_4_ gas sensor, effects of temperature, concentration of testing gas on sensitivity are discussed briefly here. The optimized performances of ZnGa_2_O_4_ gas sensors for nitric oxide (NO), NO_2_, and LPG are listed in Table 9.

#### 5.2.1. Gas Sensing Mechanism

**Figure 24:**
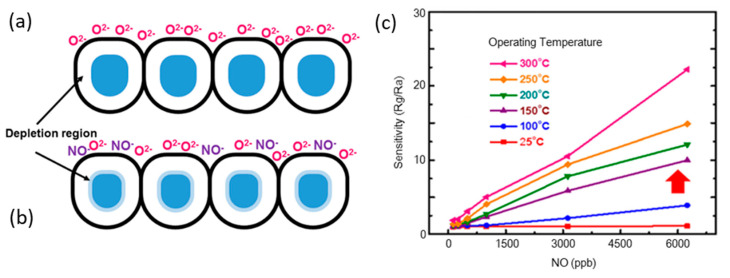
Interactions between the surface of ZnGa_2_O_4_ thin film and adsorbed molecules: (**a**) before injection of NO and (**b**) after injection of NO. (**c**) Sensitivity of ZnGa_2_O_4_ gas sensor versus different NO gas concentrations at different temperatures from 25 to 300 °C [94].

When n-type ZnGa_2_O_4_ gas sensors are exposed to air, the dangling bonds on the surface of ZnGa_2_O_4_ epilayers adsorb the atmospheric oxygen, which accompanied with electrons from the conduction band of ZnGa_2_O_4_ surface to the adsorbed oxygen molecules, becoming the forms of O^2−^_(ads)_, O^2−^_(ads)_ or O^−^_(ads)_, which is negatively charged [99]. Figure 24a shows the interactions between the surface of the ZnGa_2_O_4_ thin film and the adsorbed oxygen ions before gas injection. This mechanism creates the depletion region in the deposited ZnGa_2_O_4_ film [94] and results in an increase in resistivity [95].

Figure 24b shows the interactions between the ZnGa_2_O_4_ thin-film surface and the NO gas molecules. Because of the high electronegative property of NO, when NO gas is injected into the chamber, NO gas molecules trap the electrons from the ZnGa_2_O_4_ surface or from the adsorbed oxygen molecules and become NO^−^, as shown below [94]:

NO_(gas)_ + O_2_^−^_(ads)_ → NO^−^_(ads)_ +O_2(gas)_ (2)

NO_(gas)_ + O^−^_(ads)_ → NO^2−^_(ads)_ (3)

The resistance of the ZnGa_2_O_4_ semiconductor increases due to the abstraction of the electrons from the ZnGa_2_O_4_ surface or adsorbed oxygen molecules by NO_(gas)_. However, when ZnGa_2_O_4_ is exposed to a reducing gas like LPG, the LPG reacts with the chemisorbed oxygen and liberates the captured electrons back to the conduction band, which decreases the resistance of ZnGa_2_O_4_ [99].

#### 5.2.2. Effects on Gas Sensitivity

When the target gas is injected into the test chamber, the resistance in the target gas (R_g_) is obtained. The chamber is purged with air and the experiments are repeated. The sensitivity of gas sensors to the target gas is defined as the ratio of resistance in the air (R_a_) to that in the target gas (R_g_). NO and NO_2_ can increase the resistance to a higher value, while LPG causes the resistance to reach a lower value [99]. Therefore, the sensitivity is defined as R_g_/R_a_ for NO and NO_2_, while the sensitivity is defined as R_a_/R_g_ for LPG [99].

To find the precise operating temperature, many experiments have been done. In principle, a higher temperature could enhance gas sensitivity. There are three reasons for the rule [94]:

1. Particles originally adsorbed on the surface desorb due to high temperature, which creates more states on the ZnGa_2_O_4_ surface to react with the target gas.
2. Increasing temperature results in changing the type of adsorbed oxygen molecules from O^2−^ to O^−^ (O^−^ is more reactive than O^2−^) and helps the target gas to react very easily with O^−^.
3. Kinetic energy of the target gas is provided by high temperature, which speeds up the abstraction of the target gas on the surface of the ZnGa_2_O_4_ gas sensor.

Figure 24c shows the relationship between sensitivity and NO gas concentration, with operating temperatures from 25 to 300 °C. An obvious increase in sensitivity to NO gas with the increase in operating temperature from 100 to 150 °C (red arrow) was observed and the highest sensitivity was achieved at 300 °C. However, the highest sensitivity for NO_2_ was at 240 °C, as reported in Reference [99]. Therefore, the increasing temperature is not always necessary to achieve a higher gas sensitivity. Figure 24c shows the relationship between the different concentrations of NO and the sensitivity of the ZnGa_2_O_4_ gas sensor, which was found to be linear. This relationship was also found linear for NO_2_ [95,98], but not found linear for LPG [97].

#### 5.2.3. Gas Selectivity

A gas sensor must possess better gas selectivity so that it can detect and respond to different gases. Chen et al. reported that when the operating temperature was increased to 240 °C, the sensitivity to NO_2_ (R_g_/R_a_ = 16.2) of 5 ppm was the highest among all the corresponding values to other gases such as 50 ppm of LPG, ethanol, H_2_, and CO, respectively [99]. They all had a much higher concentration than that of NO_2_. Although the sensitivity of LPG is not as high as that of NO_2_, even ZnGa_2_O_4_ gas sensors also have a high selectivity to LPG. Jiao et al. compared LPG with CO, C_2_H_5_OH, and CH_4_ [97]. All of them are taken in the concentration of 500 ppm. The sensitivity to LPG was 10, which was much higher than that to CO, C_2_H_5_OH, and CH_4_ with sensitivities of 1.6, 2.0 and 1.4, respectively.

Wu et al. investigated the selectivity of the ZnGa_2_O_4_ gas sensor by injecting CO_2_, CO, NO, NO_2_, and SO_2_ at the same operating temperature of 300 °C [94]. The sensor detected CO_2_ and CO with poor performance. It reacted with SO_2_, but exhibited low sensitivity (1.27) against a high SO_2_ concentration (125 ppm). After comparing the gas concentration and the sensitivity, the results implied that the ZnGa_2_O_4_ gas sensor exhibits a higher selectivity to NO than NO_2_ at an operating temperature of 300 °C.

### 5.3. Phosphors

ZnGa_2_O_4_ is widely used as a phosphor host material in illumination, flat-panel displays, vacuum fluorescent displays, detectors, and in other electronic applications [101]. Sulfide phosphors emit sulfide gases such as S, SO, and SO_2_ and degrade the phosphor material during electron excitation, thus not only decreasing the luminous efficiency of the phosphor material but also decaying the performance of the cathode filament in vacuum fluorescent displays. Sulfide-based phosphors are typically more efficient than oxide phosphors under the same voltages and current densities, but degradation under electron bombardment leads to contaminate the cathode components in displays [100].

It was generally found that phosphor decomposition takes place because of contamination and the oxidizing atmosphere. Phosphors used in emission displays must have consistency at low voltages and must be resistant to Coulombic aging. The permanent loss of efficiency because of continuous electron bombardment at high current densities is the mechanism of Coulombic aging. Surface morphology, particle size, crystallinity, and homogeneous distribution of dopant activators throughout the host material are important to achieve better characteristics of phosphor particles [102].

ZnGa_2_O_4_ is a transparent semiconductor oxide and has a transparency in the near-ultraviolet region [3]. The excellent chemical stabilities of these oxide-based phosphors make them more suitable candidates than sulfide-based phosphors. ZnGa_2_O_4_ emits blue light intrinsically around 470 nm which comes from the transitions of the self-activated center of the octahedral GaO_6_ group in the spinel lattices. The Ga^3+^ ions combine with UV-generated free electrons produced in oxygen vacancies and can switch the emission to green or red when doped with activators such as Mn^2+^, Eu^3+^ [103,104].

**Figure 25:**
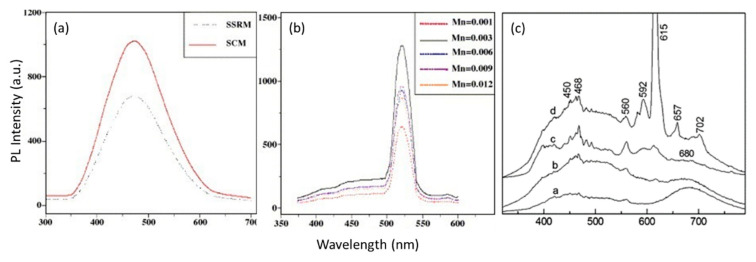
(**a**) Emission spectra of ZnGa_2_O_4_; (**b**) emission spectra of Zn_1-x_Mn_x_Ga_2_O_4_ with different Mn concentrations [105] (with copyright permission from Elsevier, 2020); (**c**) photoluminescence spectra of ZnGa_2_O_4_ powders doped with a. 4% Co; b. 4% Co, 8% Eu; c. 2% Co, 8% Eu; d. 8% Eu [106] (with copyright permission from Elsevier, 2020).

Lee et al. [105] studied the ZnGa_2_O_4_ phosphor with Mn doping prepared by solid combustion method (SCM) and solid-state reaction method (SSRM). PL spectra of ZnGa_2_O_4_ powder prepared by both the methods have been shown in Figure 25a, that shows the spectra having an intense peak at near 470 nm (blue color), having a broad range from 350 to 650 nm. As shown in Figure 25a, the spectra peak prepared by SCM has a higher intensity, due to the larger surface area than that prepared by the SSRM method. Figure 25b shows the sharp emission spectra for Zn_1-x_Mn_x_Ga_2_O_4_ phosphors where Mn has a different concentration range. The highest peak was obtained for Mn = 0.003 concentration, which shifted the emission from blue to green centered at near 513 nm.

Photoluminescence properties of ZnGa_2_O_4_ powders doped with various concentrations of Eu^3+^ and Co^2+^ were investigated by Duan et al. [106]. ZnGa_2_O_4_ is a normal spinel where Zn occupies tetrahedral sites and Ga occupies octahedral sites. However rare-earth ions may occupy both tetrahedral and octahedral sites. The photoluminescence spectra of ZnGa_2_O_4_ powders is shown in Figure 25c for the various concentrations of rare-earth ion Eu^3+^ and transition metal ion Co^2+^. Strong emission at 615 nm takes place in addition to several weak emission peaks in the red region. The luminescence at 400–500 nm was observed for the sample which is doped with 8% Eu. The spectra have various emission lines corresponding to intrashell transitions such as ^5^D_0_–^7^F_1_ (592 nm), ^5^D_0_–^7^F_2_ (615 nm), ^5^D_0_–^7^F_3_ (657 nm)_,_ and ^5^D_0_–^7^F_4_ (702 nm) of Eu^3+^ ions. Consider the sample c, as shown in Figure 25c: the emission intensity of Eu^3+^ at 615 nm decreases whereas the emission intensity of Co^2+^ ions at 680 nm increases with the increase in doping of Co^2+^ ions. These results indicate that the presence of an energy transfer from Eu^3+^ ions to Co^2+^ ions takes place. It is clearly visible that the relative intensity of the two PL peaks at 615 and 680 nm changes with the doping content of Eu and Co in the samples. These results indicate that the changing relative concentration of Eu and Co in ZnGa_2_O_4_ powders can tune the color of luminescence. The sharper spectra are usually preferred for displays that utilize all the three pure color phosphors with red, green and blue.

## 6. Conclusions

ZnGa_2_O_4_ is a spinel oxide which is attaining considerable interest due to its excellent chemical and thermal stabilities in various applications, such as field emission displays, photovoltaics, flat panel displays, vacuum fluorescent displays, phototransistors, gas sensors, UV photodetectors, and photo-catalysts, etc. [76,90,97,98,99,104,107,108,109,110,111,112,113]. In the past, ZnGa_2_O_4_ was found to be suitable to use in phosphors as a ceramic material. ZnGa_2_O_4_ is known to be an intrinsically blue emitter through the transitions of self-activated centers while considering the luminescence characteristics [33,114,115,116]. However, with the doping of Mn and Cr, this blue emission can be shifted from blue to green and red emission, respectively [73,117,118]. Continuous studies on luminescence characteristics are taking place from ceramic to thin film phosphors using various growth techniques [16,28].

Physical, electrical, optical, and mechanical properties are discussed for device performance and processing. Being a transparent oxide and wide-bandgap semiconductor, it was found to be suitable to use as a DUV photodetector. The effect of oxygen partial pressure, annealing temperature, and substrate temperature are also discussed, which require attention in order to achieve higher efficiency for the device fabrication. This material has also shown the potential as a NO_x_ gas and LPG sensor, due to the interaction of surface reactions with the adsorbed molecules [94,95,96].

Nanostructures exhibit superior properties to bulk materials, due to their large surface-volume ratio. Hence, growth from the bulk crystal to epitaxial ZnGa_2_O_4_ thin films shows the enhanced attraction of ZnGa_2_O_4_ as a functional material for device fabrication. In addition to the MSM structure for a DUV photodetector, the fundamental properties and defects associated with thin films such as oxygen vacancies, surface roughness, and dislocation have been discussed.

Recently, Chikoidze et al. [7] demonstrated that spinel ZnGa_2_O_4_ is the native p-type ternary oxide semiconductor with the widest bandgap. Their work has extended to cover the bipolar mechanisms in optoelectronics and power electronics. Since p-n junction photodetectors can have a fast response, low dark current, and high sensitivity, so future work must include the p-type conductivity of this material, ensuring the availability of the p-n junction photodetector. However, controlled growth, surface passivation, and defects associated with these devices must be overcome in the future by significant research and development, which will enhance the benefits of this material to use in the feasible applications.

Electric vehicles (EVs) and hybrid electric vehicles (HEVs) require the necessity of a highly efficient onboard AC/DC charger, and these chargers must have less size, weight, and high switching characteristics. Thermal runaway management and reduction in power consumption in harsh industrial environments lead to the necessity of low power consuming Schottky rectifiers in EVs and HEVs. Hence, Schottky rectifiers and MOSFETs based on wide-bandgap semiconductors enable the creation of smaller and better components, that can further improve the advancement of electric vehicles in industrial motors. ZnGa_2_O_4_ has an attractive application for Schottky rectifiers because of its fast switching speed in industrial motors, higher efficiency, and variable speed drives in various inductive motors-pumps, fans, compressors, and power supplies.

ZnGa_2_O_4_ has shown potential as an anode material in rechargeable batteries, due to its high electrical conductivity and high electrochemical activities [119]. Additionally, the next-generation developments in power electronics, concerning high efficiency and power density at low production cost, will make ZnGa_2_O_4_ desirable. Improvement in the efficiency of perovskite solar cells has also been observed recently by using the coating of ZnGa_2_O_4_, which indicates its future use in the upcoming devices [120].

The role of detection of partial discharges—which can produce UV-C arcs—can play an effective role in arc detection in power grids at early stages, preventing faults in electrical systems. Novel thermal management must be taken into consideration for the design of power devices. Stability ensures the smooth operation of the device; hence, identification of dominant defects—such as oxygen vacancies and their role in residual conductivity relative to extrinsic impurities associated with the device—must be figured out. The continued development of high-quality bulk crystals and thin films is necessary to fabricate devices with the appropriate synthesis techniques that will ensure the regimes of stability for the next-generation power devices.

## Figures and Tables

**Table 1:** Comparison of various wide-bandgap semiconductor materials properties (the data are from [37] except for ZnGa_2_O_4_).

	4H-SiC	GaN	β-Ga_2_O_3_	ZnGa_2_O_4_
**Bandgap, E_g_ (eV)**	3.25	3.4	4.85	4.325 (indirect) 4.570 (direct) [38]
**Dielectric constant, *ε***	9.7	9	10	10.4 [39]
**Breakdown field, E_C_ (MV/cm)**	2.5	3.3	8	-
**Electron mobility, μ (cm^2^/V·s)**	1000	1250	300	100 [6,40]
**Saturation velocity, v_s_ (10^7^ cm/s)**	2	2.5	1.8–2	-
**Thermal conductivity λ (W/cm·K)**	4.9	2.3	0.1–0.3	0.22 [38]

**Table 2:** Summary of ZnGa_2_O_4_ bulk grown methods along with their characteristics.

**Method**	Solid State	Flux grown	Czochralski	LHPG	Hydrothermal
**Raw material**	ZnO and Ga_2_O_3_				ZnSO_4_.10H_2_O and Ga_2_O_3_
**Morphology**	Porous natured rods	Spinel		Fibers	Cuboids
**Crystal dimension**	1–5 µm	3–10 mm	5 mm	-	35–60 nm
**Temperature** **(°C)**	1000	1000–1500	600–800	1100	160–200
**Bandgap (eV)**	4.74	4.0	4.6	-	-
**Lattice constant (Å)**	8.37	8.332	8.333	-	-
**Reference**	[69]	[57,58]	[38]	[64]	[70]

**Table 3:** Comparisons of photoluminescence (PL) peaks and crystalline orientations with different process parameters in the sputtering process.

**Substrate**	c-plane sapphire	Si(100)	c-plane sapphire	Si(100)
**Working Chamber pressure (Torr)**	5 × 10^−3^		5 × 10^−3^	4 × 10^−3^
**RF power (W)**	150			
**Substrate temperature (°C)**	From 200 to 600		400	200–600
**Annealing temperature (°C)**	-		500–900	700–900
**Characteristic Peaks** **in XRD Pattern**	(111), (311), (222), (400), (511)	(111), (220), (311), (222), (400), (511)	(220), (311), (222), (400), (511), (440)	(222), (220), (311)
**Luminescent Peak (nm)**	340, 417, 512	512	340, 520	470–360
**Reference**	[78]		[74]	[33]

**Table 4:** Comparisons of PL peaks and crystalline orientations with different process parameters in the pulsed laser deposition (PLD) process.

**Substrate**	Si(100)	MgO (100)	Al_2_O_3_ (0001)	Si(100), Al_2_O_3_(0001), MgO(100)	(00.1) Sapphire
**Substrate temperature (°C)**	550	650–730	450, 550, and 650		650, 700, 750, 850
**Oxygen pressure**	50–300 mTorr	0–130 mTorr	0.1, 0.2, 0.3 Torr	100 mTorr	1.6 Pa
**Annealed temperature (°C)**	From 550 to 700	-	-		-
**Characteristic Peaks in XRD Pattern**	(111), (220), (311), (222), (400)	(400)	Si(100): (111), (220), (311), (222), (400), (511) Al_2_O_3_(0001): (111), (311), (222), (400), (511) MgO(100): (400)		(111), (222), (333), (444)
**PL peak (nm)**	From 460 to 370	479	460		-
**Reference**	[72]	[79]	[71]	[80]	[81]

**Table 5:** Cathodoluminescence (CL) peaks and crystalline orientations with different process parameters for mist chemical vapor deposition (CVD) and metal-organic CVD (MOCVD).

**Process Method**	Mist CVD	MOCVD
**Substrate**	(100)MgAl_2_O_4_	c-plane (002)sapphire
**Precursors**	Concentrations: 0 ≤ (Zn)/(Ga) ≤ 10.0 (Zn) and (Ga): zinc and gallium Acetylacetonate	Flow rates: TEGa: 50 sccm O_2_: 200 sccm DEZn: 10, 30, 40, 50, 60 sccm
**Grown temperature**	From 400 to 800 °C	-
**Crystalline orientation of** **ZnGa_2_O_4_**	(400), (800)	(111), (222), (333)
**Crystalline orientation of** **Ga_2_O_3_**	(400), (800)	(-201), (-402), (-603)
**CL peak**	365, 428 and 495 nm	Main: 332 nm Weak: 236, 499 nm
**Reference**	[84]	[76]

**Table 6:** Elemental compositions (Ga, Zn, O, and C), the ratios of Ga/Zn and O/(Zn + Ga) for ZnGa_2_O_4_ films prepared at various DEZn flow rates of 10−60 sccm by using X-ray photoelectron spectroscopy (XPS) measurements [76].

DEZn Flow Rate	Ga (Atom %)	Zn (Atom %)	O (Atom %)	C (Atom %)	Ga/Zn	O/(Zn + Ga)
10 sccm	46.4	2.0	50.7	0.9	23.2	1.05
30 sccm	39.2	8.1	51.3	1.4	4.84	1.08
40 sccm	38.7	10.9	49.5	0.9	3.55	0.99
50 sccm	31.5	10.5	54.3	3.7	3	1.29
60 sccm	26.9	10.0	44.2	18.9	2.69	1.20

**Table 7:** Process parameters of the four references about photodetectors. ‘RT’ represents room temperature.

**Method**	MOCVD		sputtering	
**Substrate**	c-plane (0001) sapphire		quartz	c-plane sapphire
**Substrate temperature (°C)**	650		-	From RT to 600
**Precursors**	DEZn:40 sccm, TEGa:50 sccm, O_2_(99.999%): 200 sccm		-	
**Annealing temperature (°C)**	800	700, 800, 900	100, 200, 300	From 500 to 900
**Reference**	[87]	[92]	[20]	[93]

**Table 8:** Optimized performances of photodetectors in the four references.

**Light Irradiation (nm)**	230		260	240
**Applied bias (V)**	20	5	10	5
**Annealed temperature (°C)**	-	800	200	700
**Responsivity (A/W)**	5.77	86.3	0.203	2.53
**Photo/dark current ratio**	4.68 × 10^4^	~10^7^	~10^9^	3.77 × 10^4^
**Rise time, decay time (s)**	0.96, 0.34	<1	13, 2	4.5, 0.2
**Reference**	[87]	[92]	[20]	[93]

**Table 9:** Optimized performances of ZnGa_2_O_4_ gas sensors.

**Test gas**	NO	LPG	LPG	NO_2_
**Concentration (ppm)**	1	500	50	5
**Temperature (°C)**	300	410	340	240
**Sensitivity**	5.03	10	7.9	16.2
**Reference**	[96]	[99]	[100]	[100]
